# *Bifidobacterium Pseudolongum‐*Derived Inosine Mitigates Polystyrene Nanoplastics‐Induced Hepatic Injury by Inhibiting the Polarization of M1 Macrophages

**DOI:** 10.1002/advs.202522510

**Published:** 2026-07-06

**Authors:** Kaikai Zhang, Yuchuan Chen, Jiayuan Wan, Jianzheng Yang, Lijian Chen, Qingyuan Li, Anding Zhou, Nian Zhou, Jiuyang Ding, Qi Wang, Jian Sun

**Affiliations:** ^1^ Department of Infectious Diseases Nanfang Hospital Southern Medical University State Key Laboratory of Multi‐Organ Injury Prevention and Treatment Key Laboratory of Infectious Diseases Research in South China Ministry of Education Guangdong Provincial Key Laboratory for Prevention and Control of Major Liver Diseases Guangdong Provincial Clinical Research Center for Viral Hepatitis Guangdong Institute of Hepatology Guangdong Provincial Research Center for Liver Fibrosis Engineering and Technology Southern Medical University Guangzhou China; ^2^ Department of Gastroenterology Shunde Hospital of Southern Medical University Southern Medical University Foshan China; ^3^ Guangzhou Key Laboratory of Forensic Multi‐Omics for Precision Identification School of Forensic Medicine Southern Medical University Guangzhou China

**Keywords:** *Bifidobacterium pseudolongum*, gut‐liver axis, hepatic injury, macrophage, nanoplastics

## Abstract

Nanoplastics (NPs) exposure can cause severe hepatic injuries. Gut microbiota is considered a contributing factor to multiple hepatic injuries. However, its role in NPs‐induced hepatic injuries remains unclear, and microbial intervention strategies are required. Our results reveal that oral exposure to polystyrene NPs reduces gut probiotic *Bifidobacterium pseudolongum* (*B.p*) and its metabolite inosine. Gut microbiota from NPs‐administered mice partially reproduces NPs‐related impairment of gut homeostasis and hepatic injury in recipient mice. Moreover, *B.p* colonization improves NPs‐induced gut homeostasis impairment and hepatic injury, and its protective effects are reproduced by supplementation with inosine. Mechanically, *B.p* colonization increases hepatic level of inosine and subsequently normalizes the expression of its target A2AR. Meanwhile, increased inosine inhibits the miR155/SOCS1/NF‐κB pathway and represses NPs‐induced M1 macrophage polarization. CGS21680, an agonist of A2AR, effectively represses lipopolysaccharide (LPS)‐induced M1 macrophage polarization and inhibits the miR155/SOCS1/NF‐κB pathway in vitro. Further, miR155 knockout inhibits NPs‐induced M1 macrophage polarization, but does not influence the suppression of NPs on A2AR. These findings suggest that *B.p*‐derived inosine can repress NPs‐induced M1 macrophages polarization by inhibiting the miR155/SOCS1/NF‐κB pathway via targeting A2AR. Altogether, this study further clarifies the role of gut microbiota in NPs‐induced hepatic injury and provides a potential microbial therapeutic strategy.

## Graphical abstract

Probiotic *B.p* colonization elevated gut‐derived inosine level in the liver, while elevated inosine activated A2AR and subsequently blocked NPs‐induced polarization of M1 macrophages by repressing the miR155/SOCS1/NF‐κB pathway. This reduced the release of inflammatory cytokines and thereby, mitigated NPs‐induced hepatic injury.

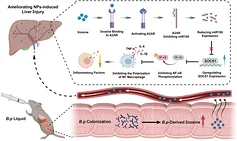

## 1 Introduction

The widespread use of plastic products has caused severe environmental problems globally [1]. During the production and natural weathering of plastic, a large number of small particles are generated, posing a great threat to public health [2]. Microplastics (MPs, diameter ˂ 5 mm) and nanoplastics (NPs, diameter ˂ 1 µm) can accumulate in the human through the food chain and are closely associated with various diseases [3]. As the largest organ of drug metabolism, the liver is vulnerable to MPs and NPs exposure [4, 5]. It has also been reported that the liver is one of the major organs for MPs and NPs accumulation; meanwhile, the small size of NPs allows them to pass through biological barriers more easily than MPs [4, 6]. In fact, MPs and NPs have been well established to induce hepatic inflammation, trigger oxidative stress, promote fibrosis, disrupt lipid metabolism, and cause other adverse effects [7, 8, 9]. A recent study revealed that daily bottled water contained approximately 2.4×10^5^ MPs and NPs per liter, with NPs accounting for over 90% [10]. However, the detailed mechanisms underlying NPs‐related hepatic injury are still undisclosed.

In the past decades, gut microbiota has been well‐recognized for its crucial role in multiple liver diseases [11, 12]. Numerous studies have demonstrated that NPs can disturb gut microbiota and cause severe hepatic injury. Recently, Xia et al. discovered that gut microbiota contributed to NPs‐induced hepatic injury [13]. Our previous study revealed that NPs‐related gut microbiota dysbiosis exacerbated the aflatoxin B1‐induced hepatic injury [14]. These indicate that gut microbiota is a vital contributor to NPs‐induced hepatic injury, while the detailed microbial mechanisms remain unclarified.

As components of innate immunity, macrophages are the primary phagocytic cells for foreign bodies and serve as important modulators of the inflammatory response [15, 16]. Macrophages have exhibited a close association with NPs‐induced biological toxicity. In vitro, NPs were found to polarize RAW264.7 cells (a mouse macrophage cell line) toward the M1 type at an appropriate concentration [17]. Moreover, NPs could promote the infiltration of macrophages and induce M1 polarization phenotype, leading to testicular inflammation [18]. Notably, gut microbiota has been observed to modulate macrophage‐related biological function. Gut microbiota metabolite valeric acid could drive the self‐renewal of intestinal stem cells by niche enteric serotonergic neurons in a macrophage‐dependent manner [19]. Moreover, gut microbiota Odoribacteraceae‐derived postbiotic isoallolithocholic acid was identified to induce marco immunosuppressive macrophages, limiting excessive inflammation at the gateway of the liver [20]. These studies emphasize the modulation of gut microbiota on macrophage polarization. However, the relationship between gut microbiota and NPs‐related M1 macrophage polarization, as well as the existence of functional microbiota protecting against NPs‐induced hepatic injury, remains unknown.

MicroRNA (miRNA) is a class of small non‐coding RNAs with 19–23 nucleotides, and exerts the vital regulatory effects in multiple biological processes, including immune response, cell proliferation, differentiation, and tissue development [21, 22, 23, 24, 25]. MicroRNA‐155 (miR155) is highly conserved in mammals and is one of the most important miRNAs in inflammatory response [26, 27]. miR155 has been reported to be highly expressed in macrophages and can modulate macrophage polarization [28]. Jiang et al. found that in sepsis‐related acute lung injury, miR155 promoted inflammatory response by triggering M1 macrophage polarization via negatively regulating SOCS1 expression [29]. Additionally, miR155 knockout could suppress non‐alcoholic fatty liver disease‐induced recruitment of macrophage and alleviate liver inflammation by inhibiting Kupffer cell polarization toward M1 [30]. Given these, we hypothesize that miR155 signaling could be involved in NPs‐induced polarization of M1 macrophages, which may affect hepatic injury.

In this study, we established a 28‐day repeated NPs exposure model to induce hepatic injury and investigated the underlying microbial mechanisms using fecal microbiota transplantation. 16S rRNA sequencing combined with non‐targeted metabolomics was employed to identify potential functional microbiota. Moreover, *B.p* colonization and supplementation with its metabolite inosine were identified as effective strategies for alleviating NPs‐induced hepatic injury. Additionally, miR155 knockout mice and A2AR agonist CGS21680 were used to investigate the mechanisms underlying the protective effect of inosine.

## 2 Results

### 2.1 NPs Induced Systemic Inflammation and Promoted the Polarization of Hepatic M1 Macrophages

In this study, mice were orally administered with low‐dose NPs (20 mg kg^−1^, L‐NPs), high‐dose NPs (40 mg kg^−1^, H‐NPs), and high‐dose MPs (40 mg kg^−1^, H‐MPs), respectively, to evaluate the dose‐ and size‐dependent hepatotoxic effects of plastic particles (Figure 1A). Fluorescence imaging revealed that, after oral exposure for 24 h, both MPs and NPs mainly distributed in the intestine, with NPs exhibiting greater intestinal retention compared to MPs (Figure 1B). During the 28 d of administration, mice exposed to NPs presented a dose‐dependent reduction in the rate of body weight gain compared to the sterile phosphate‐buffered saline (PBS) group, whereas exposure to H‐MPs did not significantly affect body weight gain (Figure 1C), hinting that NPs may induce more adverse health effects than MPs. In terms of systemic inflammation, H‐NPs presented a stronger proinflammatory effect compared to L‐NPs and H‐MPs exposure, as evidenced by the highest levels of serum LPS and inflammatory cytokines tumor necrosis factor alpha (TNF‐α), interleukin‐6 (IL‐6), and interleukin‐1β (IL‐1β) among the groups (Figure 1D). Meanwhile, the liver function indexes alanine aminotransferase (ALT) and aspartate aminotransferase (AST) were significantly elevated following exposure to L‐NPs, H‐NPs, and H‐MPs, with H‐NPs showing the highest levels among groups (Figure 1E). Moreover, in contrast to exposure to L‐NPs, both H‐NPs and H‐MPs obviously increased the liver weight and liver/body weight ratio in mice, with no significant differences observed between these two groups (Figure S1A; Figure 1E). Histomorphological analysis indicated that the H‐NPs group displayed a greater infiltration of inflammatory cells in the liver compared to the L‐NPs and H‐MPs groups (Figure 1F). Regarding pro‐fibrotic effects, H‐NPs induced a larger picrosirius red (PSR)‐ and α‐smooth muscle actin (α‐SMA)‐positive area in the liver than L‐NPs exposure, while no significant differences were observed between the H‐NPs and H‐MPs groups (Figure 1F,G). These findings suggest that the hepatic injury effects of plastic particles are dependent on both dose and size. Thus, a dose of 40 mg kg^−1^ NPs (H‐NPs) was adopted to induce hepatic injury in the subsequent study. Given the crucial role of macrophages in inflammatory response and fibrosis, we determined the polarization state of macrophages. Immunohistochemistry (IHC) and Western Blotting (WB) results showed that H‐NPs increased the expression of protein F4/80 and CD86 in the liver (Figure 1H–K), indicating the recruitment of macrophages and M1 polarization. Additionally, the miR155/SOCS1/NF‐κB pathway was activated by H‐NPs exposure, as demonstrated by the upregulation of miR155 and p‐NF‐κB/NF‐κB ratio, as well as the reduction of SOCS1 (Figure 1J–L). It has been reported that the miR155/SOCS1/NF‐κB pathway could be activated by LPS and was involved in the modulation of M1 macrophage polarization [29]. Accompanied by the polarization of M1 macrophages, we observed that H‐NPs significantly increased the levels of fibrosis‐related proteins α‐SMA and Col1a1 (Figure 1J–L), as well as inflammatory cytokines TNF‐α, IL‐6, and IL‐1β at the mRNA level (Figure 1L). These results indicate that NPs can induce hepatic inflammation and fibrosis, which may be attributed to M1 macrophage polarization.

**FIGURE 1:**
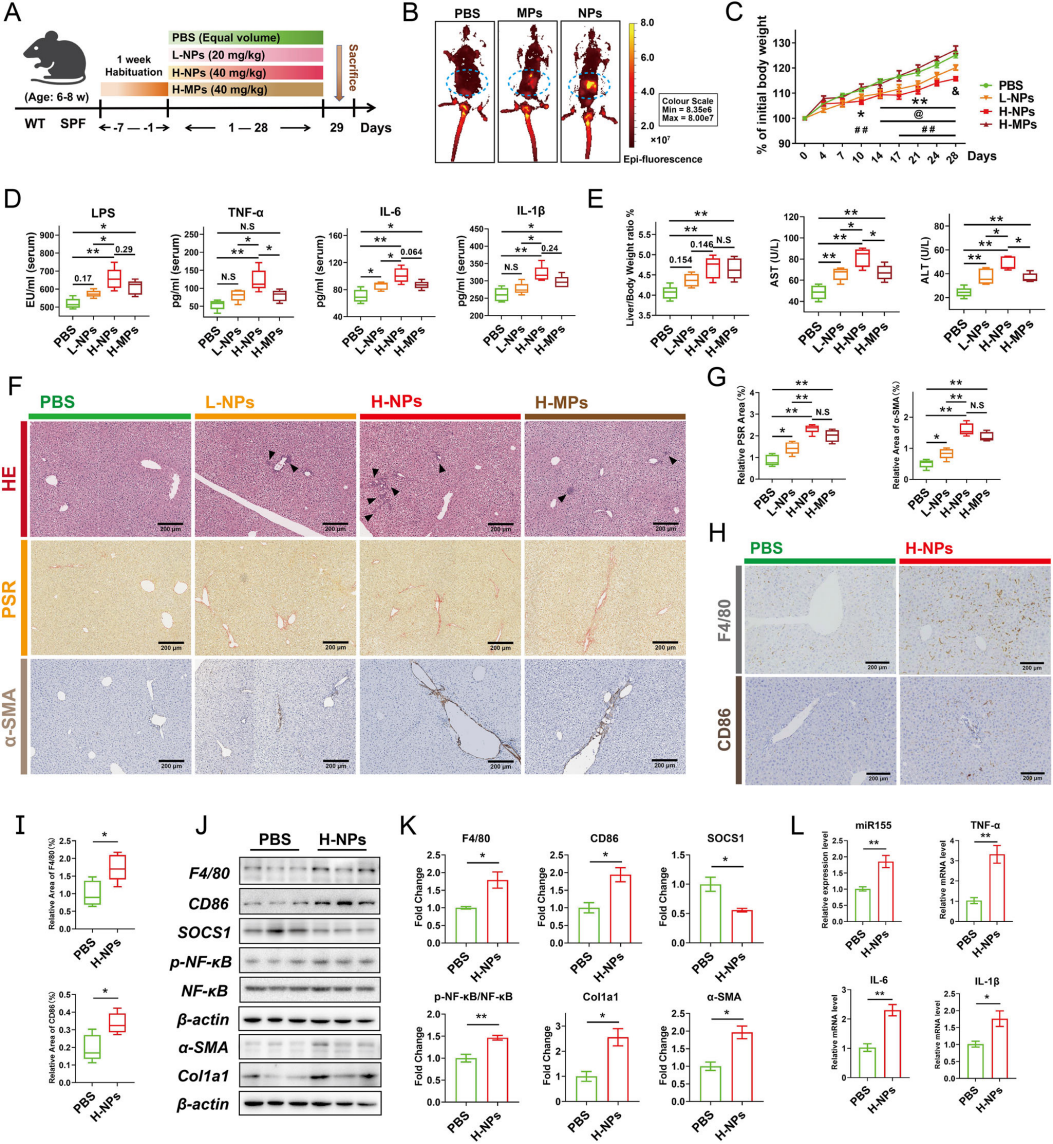
NPs stimulated systemic inflammation and facilitated M1 macrophage polarization in the liver. (A) Procedure for constructing the NPs and MPs exposure model in mice. (B) Fluorescence imaging of NPs and MPs distribution in vivo. (C) Alteration in the percentage of initial body weight during PBS, L‐NPs, H‐NPs, and H‐MPs administration. Two‐way ANOVA was performed for statistical comparisons across the groups. *p‐value ˂ 0.05, **p‐value ≤ 0.01 (H‐NPs vs. PBS); ^&^ *p*‐value ˂ 0.05 (H‐NPs vs L‐NPs); ^##^ *p*‐value ≤ 0.01 (H‐NPs vs H‐MPs); ^@^ *p‐*value ˂ 0.05, (L‐NPs vs PBS). (D) Alterations in inflammatory cytokines: LPS, TNF‐α, IL‐6, and IL‐1β levels in the serum. (E) Alterations in the liver/body weight ratio and liver function indexes: ALT and AST. (F, G) Representative images of HE, PSR, and IHC staining for α‐SMA in the liver, and the corresponding quantification of PSR‐positive area and IHC staining. Scale bar = 200 µm. (H, I) Representative images of IHC staining for F4/80 and CD86 in the liver; and the corresponding quantification of IHC staining. (J, K) WB analysis of F4/80, CD86, SOCS1, p‐NF‐κB, NF‐κB, α‐SMA, and Col1a1 expression in the liver, normalized to β‐actin. (L) RT‐qPCR analysis for the expression of mRNA TNF‐α, IL‐6, and IL‐1β, as well as miR155 in the liver; β‐actin and U6 genes were used as the internal controls for mRNA and miRNA, respectively. Student's t‐test was used for comparison between the two groups, while one‐way ANOVA with post hoc Tukey test was employed for comparisons among multiple groups. All Data were expressed as mean ± SEM, *p‐value ˂ 0.05, **p‐value ≤ 0.01, *n* ≥ 3.

### 2.2 NPs Damaged the Gut Homeostasis by Inducing Colonic Inflammation, Impairing the Intestinal Barrier, Disturbing the Gut Microbiota, and Reshaping the Fecal Metabolome

Considering the close association between gut homeostasis and the liver, we investigated the alterations in gut homeostasis after H‐NPs exposure. Compared to the PBS group, mice in the H‐NPs group exhibited a shorter length of colon (Figure 2A), hinting the occurrence of colonic inflammation. Hematoxylin and eosin (HE) staining showed that H‐NPs caused a widening of crypt space, whereas alcian blue (AB) and periodic acid‐Schiff (PAS) staining indicated fewer AB‐positive (AB^+^) and PAS‐positive (PAS^+^) goblet cells after H‐NPs exposure (Figure 2B). Meanwhile, transmission electron microscopy (TEM) examination displayed that H‐NPs reduced the length of intestinal microvilli and disrupted the microvilli arrangement compared to the PBS group (Figure 2B). Correspondingly, H‐NPs decreased the expression of tight junction proteins zonula occludens protein 1 (ZO‐1), Occludin, and Claudin‐5 (Figure 2C,D), and increased the mRNA levels of TNF‐α, IL‐6, and IL‐1β in the colon (Figure 2E). These results indicated the impairment of the intestinal barrier and the stimulation of colonic inflammation. Moreover, H‐NPs resulted in a significant increase of TUNEL‐positive cells in the colon (Figure 2C), suggesting the pro‐apoptotic effect of H‐NPs. Next, RNA sequencing was performed to reveal the detailed mechanisms underlying H‐NPs‐induced colonic injury. H‐NPs induced 646 differential expression genes (DEGs), including 574 upregulated and 72 downregulated DEGs (Figure 2F). Gene Ontology (GO) Enrichment analysis revealed that DEGs were primarily enriched in gut microbiota‐related biological processes (Figure 2G). Go Annotation analysis was further performed on DEGs according to biological process, cellular component, and molecular function (Figure 2H). These results indicate that NPs stimulate colonic inflammation and impair the intestinal barrier, which may be related to gut microbiota.

**FIGURE 2:**
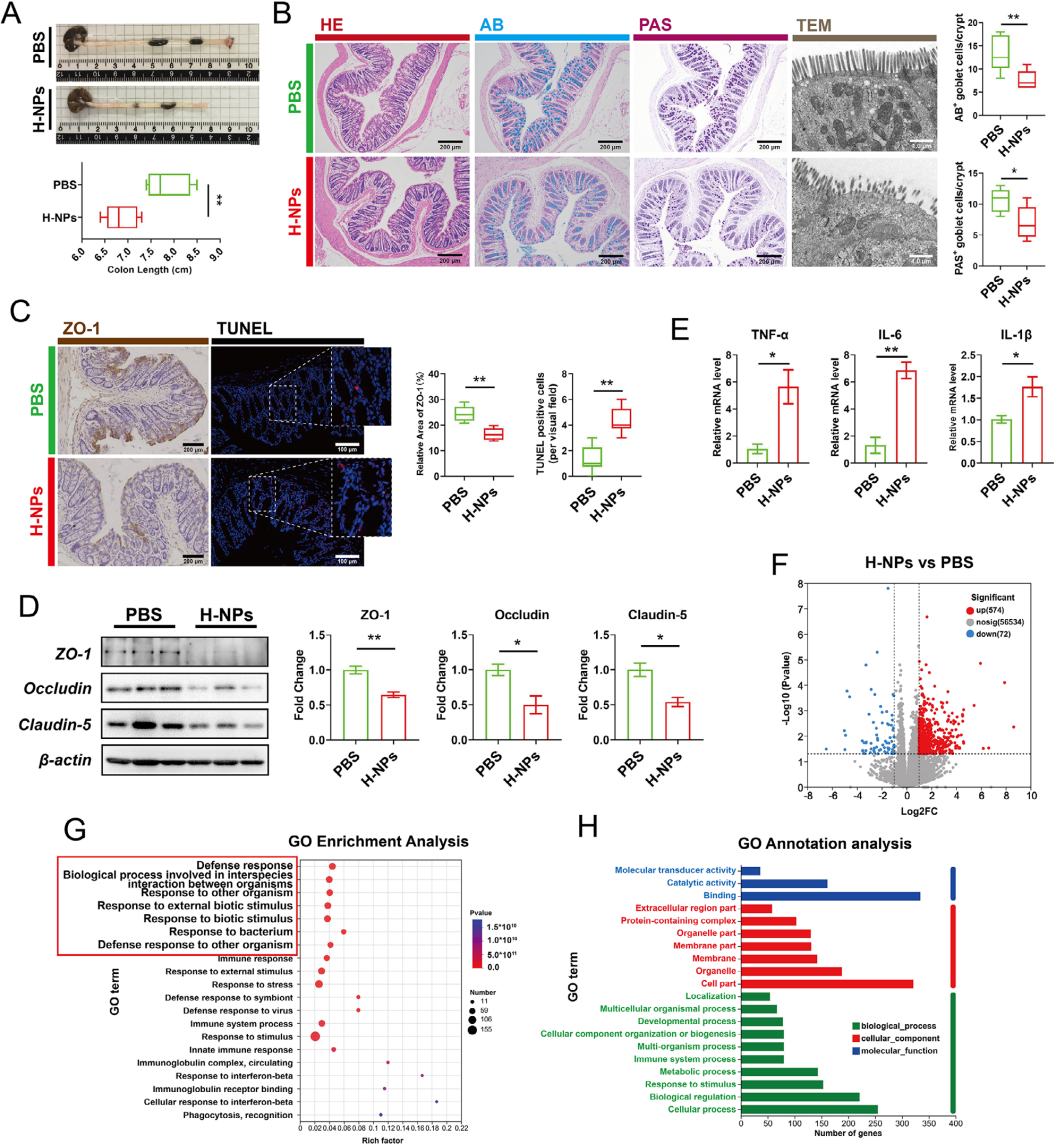
NPs impaired the intestinal barrier and stimulated colonic inflammation. (A) Representative images of the colon after PBS and H‐NPs administration and changes in colonic length. (B) Representative images of HE, AB, and PAS staining (scale bar = 200 µm) and TEM (scale bar = 1 µm) of the colon, and the corresponding quantification of AB^+^ and PAS^+^ goblet cells per crypt. (C) Representative images of IHC staining for ZO‐1 (scale bar = 200 µm) and TUNEL staining (scale bar = 100 µm), and corresponding quantification of the relative area of ZO‐1 and the number of TUNEL‐positive cells per field. (D) WB analysis of ZO‐1, Occludin, and Claudin‐5 expression in the colon, normalized to β‐actin. (E) RT‐qPCR analysis for the relative expression of mRNA TNF‐α, IL‐6, and IL‐1β in the colon; β‐actin gene was used as an internal control. (F) Volcano plot of H‐NPs‐induced DEGs in the colon. (G) GO Enrichment analysis of H‐NPs‐induced DEGs. (H) GO Annotation analysis of H‐NPs‐induced DEGs according to molecular function (blue bars), cellular component (red bars), and biological process (green bars). All data were analyzed using unpaired Student's t‐test and expressed as mean ± SEM, *p‐value ˂ 0.05, **p‐value ≤ 0.01, *n* ≥ 3.

Next, 16S rRNA‐seq was used to investigate the alterations in the gut microbiota after H‐NPs exposure. Rank‐abundance curves exhibited no significant differences in operational taxonomic unit (OTU) abundance or evenness among the samples (Figure 3A). In alpha diversity, H‐NPs induced the higher ACE and Shannon indexes than the PBS group, indicating an increase in microbial richness and diversity (Figure 3B). Principal coordinate analysis (PCoA) showed that samples in the H‐NPs group were apparently separated from those in the PBS group, indicating alterations in microbial composition after H‐NPs exposure (Figure 3C). Meanwhile, H‐NPs deteriorated the health of the microbial community, as demonstrated by the lower Gut Microbiome Health Index (GMHI; Figure 3D). At the phylum level, the microbial structure of the H‐NPs group was obviously different from that of the PBS group (Figure 3E). At the species level, we observed that among the top 10 differential microbiota, the abundance of probiotics *Akkermansia muciniphila* and *Bifidobacterium pseudolongum* (*B.p*) was significantly decreased after H‐NPs exposure (Figure 3F). In comparison to the PBS group, exposure to H‐NPs resulted in a decreased proportion of probiotics and an increased proportion of pathogens. Consequently, the ratio of probiotics/pathogens was significantly reduced after H‐NPs exposure (Figure S1B). Furthermore, LEfSe Cladogram was conducted to visualize the taxonomic groups and affiliations of differential microbiota from the phylum to genus levels (Figure 3G).

**FIGURE 3:**
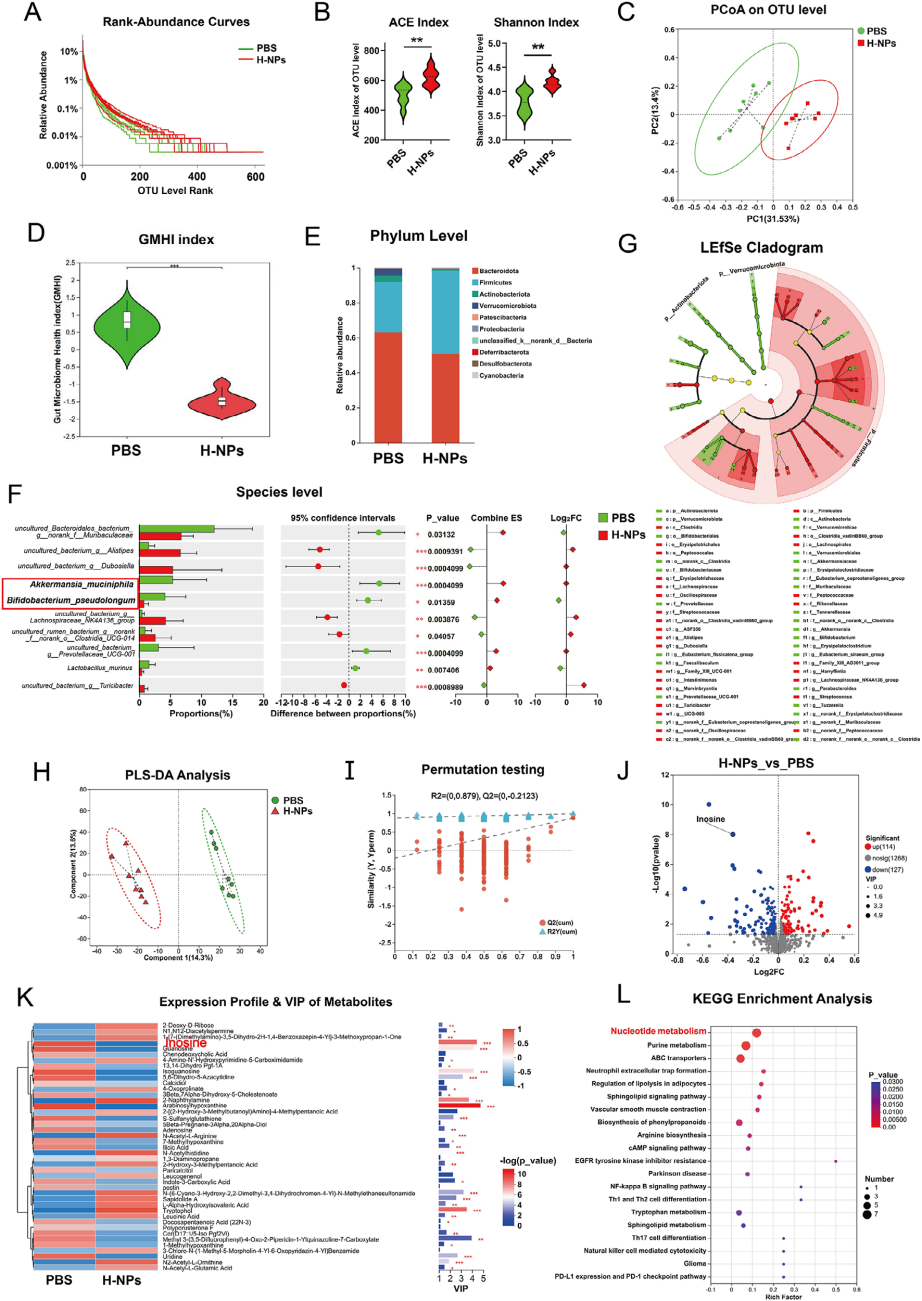
NPs disrupted gut microbiota and reshaped the fecal metabolome. (A) Rank‐abundance curve of all samples. Green curves represented the samples in the PBS group, while red curves denoted the samples in the H‐NPs group. (B) Alterations in alpha diversity after H‐NPs exposure, as demonstrated by ACE and Shannon indexes. (C) PCoA analysis of the microbial components. Samples in the PBS group were represented by green dots, and H‐NPs samples were indicated by red squares. (D) Alterations in gut microbiome health index between the two groups. (E) Alterations in relative microbial abundance at the phylum level. (F) The top 10 differential microbiota between the two groups at the species level. (G) LEfSe Cladogram indicated the phylogenetic distribution of differential microbiota correlated with the PBS and H‐NPs groups. The circles from the inside to the outside represented the different taxonomic levels from genus to phylum. Differential microbiota between the two groups was filtered using the Wilcoxon rank‐sum test with a 95% confidence interval. (H, I) PLS‐DA score plots and the corresponding permutation testing based on the LC/MS metabolite profiles of fecal samples in the PBS and H‐NPs groups. (J) Volcano plot of H‐NPs‐induced differential metabolites in fecal samples. (K) Heatmap analysis for the relative expression of differential metabolites and the corresponding VIP values. (L) KEGG enrichment analysis for the differential metabolites (top 20 enriched pathways). All data were analyzed using unpaired Student's t‐test and expressed as mean ± SEM, *p‐value ˂ 0.05, **p‐value ≤ 0.01, *n* ≥ 6.

There is accumulating evidence that microbiota‐derived metabolites mediate interactions between gut microbiota and the liver [31, 32, 33]. Therefore, alterations in the fecal metabolome were profiled using the untargeted metabolomics. Partial least squares‐discriminant analysis (PLS‐DA) displayed a significant difference in component between the H‐NPs and PBS groups (Figure 3H), while permutation testing further verified its high reliability: R2 = (0, 0.879), Q2 = (0, ‐0.2123) (Figure 3I). H‐NPs induced 241 differential metabolites (114 upregulated and 127 downregulated), of which only 43 metabolites could be annotated (Figure 3J,K). Corresponding to the decrease in *B.p* abundance, the concentration of its metabolite inosine was also decreased after H‐NPs exposure (Figure 3J,K). KEGG enrichment analysis revealed that inosine‐related Nucleotide metabolism was identified as the most significantly enriched pathway (Figure 3L). Furthermore, the serum and liver concentrations of inosine were significantly decreased after H‐NPs exposure, accompanied by the lower expression of its target protein adenosine receptor A2a (A2AR) in the liver (Figure S2A,B). Additionally, we assessed the abundance of fecal *B.p*, as well as the concentrations of inosine in serum and liver across the PBS, L‐NPs, H‐NPs, and H‐MPs groups. We found that mice exposed to H‐NPs and H‐MPs exhibited the lowest abundance of *B.p* among the groups, with no statistical differences between them (Figure S1C). Meanwhile, L‐NPs, H‐NPs, and H‐MPs were found to significantly lower serum and liver concentrations of inosine in mice, with H‐NPs causing the lowest concentrations among the groups (Figure S1D). These results imply that inosine may be the key metabolite in interactions between the gut microbiota and the liver.

In addition, the potential toxic effects of H‐NPs on other major organs were also evaluated. We found that, compared to the PBS group, H‐NPs elevated serum levels of myocardial biomarkers lactate dehydrogenase (LDH) and creatine kinase (CK) (Figure S3A). Histological analyses showed that H‐NPs promoted inflammatory cell infiltration in the cardiac tissue without increasing the Masson‐positive area (Figure S3C), suggesting that H‐NPs may promote cardiac inflammation and myocardial injury. Moreover, renal function indexes UREA and creatinine (CREA) were also significantly elevated after H‐NPs exposure. This was accompanied by increased infiltration of inflammatory cells and a larger Masson‐positive area in the kidney (Figure S3B,C), indicating that H‐NPs may induce renal inflammation and fibrosis. Similar pro‐inflammatory and pro‐fibrotic effects of H‐NPs were also observed in the lung, as demonstrated by increased inflammatory cell infiltration and Masson‐positive area (Figure S3C). In addition, exposure to H‐NPs did not result in abnormal histomorphological changes in the spleen, as evidenced by the results of HE and Masson staining (Figure S3C).

### 2.3 Gut Microbiota Partially Reproduced NPs‐Induced Damage of Gut Homeostasis and Hepatic Injury

To confirm the crucial role of gut microbiota, gut microbiota from PBS‐ or H‐NPs‐administered mice were transplanted into ABX‐pretreated recipients (FMT‐C or FMT‐N, Figure 4A). Compared to the FMT‐C group, mice in the FMT‐N group presented a lower rate of body weight gain from day 18 (Figure 4B). Meanwhile, H‐NPs‐induced shortening of colonic length was reproduced by FMT‐N administration (Figure 4C). FMT‐N‐administered mice exhibited fewer AB^+^ and PAS^+^ goblet cells than those in the FMT‐C group (Figure 4D,E). Additionally, FMT‐N reduced and shortened the intestinal microvilli, and disrupted their arrangement (Figure 4D). In terms of tight junction proteins, the expression levels of ZO‐1, Occludin, and Claudin‐5 were obviously decreased by FMT‐N in comparison with the FMT‐C group (Figure 4D–F), indicating the impairment of the intestinal barrier. Moreover, gut microbiota from H‐NPs‐administered mice induced apoptosis and inflammation in the colon of recipients, as demonstrated by the increased number of TUNEL‐positive cells and the elevated mRNA levels of TNF‐α and IL‐1β (except for IL‐6) (Figure 4D,E,G). These results suggest that gut microbiota contribute to NPs‐induced damage of gut homeostasis.

**FIGURE 4:**
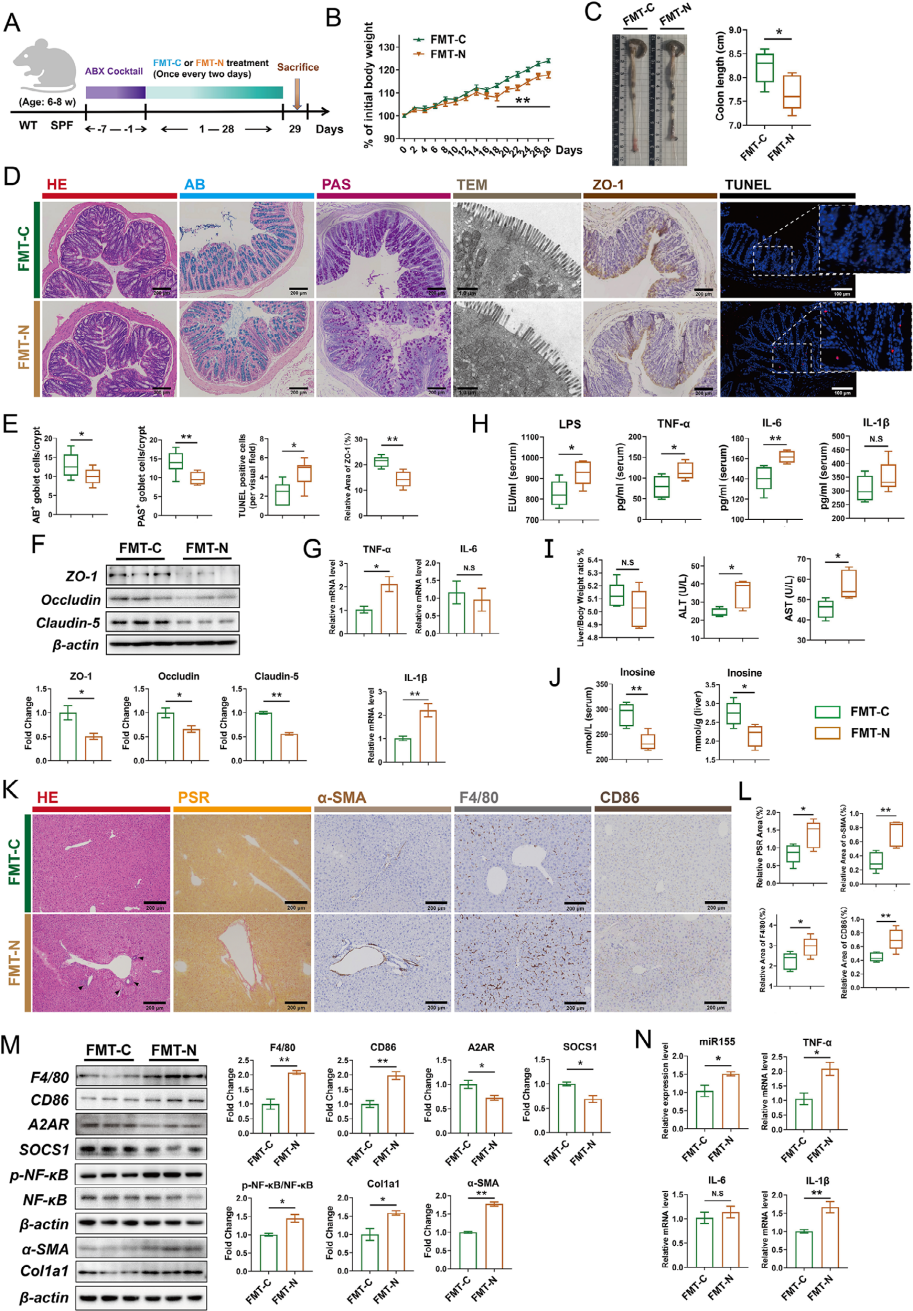
Gut microbiota mediated NPs‐induced gut homeostasis impairment and M1 macrophage polarization. (A) Procedure for constructing the FMT model in mice. (B) Changes in the percentage of initial body weight during FMT‐C and FMT‐N administration. (C) Representative images of the colon after FMT‐C and FMT‐N administration and alterations in colonic length. (D, E) Representative images of HE, AB, PAS, and IHC staining for ZO‐1 (scale bar = 200 µm), TEM (scale bar = 1 µm), and TUNEL staining (scale bar = 100 µm) of the colon, and the corresponding quantification of AB^+^ and PAS^+^ goblet cells per crypt, the relative area of ZO‐1, and the number of TUNEL‐positive cells per field. (F) WB analysis of ZO‐1, Occludin, and Claudin‐5 expression in the colon, normalized to β‐actin. (G) RT‐qPCR analysis for the relative expression of mRNA TNF‐α, IL‐6, and IL‐1β in the colon; β‐actin gene was used as the internal control. (H) Alterations in inflammatory cytokines: LPS, TNF‐α, IL‐6, and IL‐1β levels in the serum between the FMT‐C and FMT‐N groups. (I) Alterations in liver/body weight ratio and liver function indexes: ALT and AST. (J) Alterations in inosine levels in the serum and liver. (K, L) Representative images of HE, PSR, and IHC staining for α‐SMA, F4/80, and CD86 in the liver; the corresponding quantification of PSR‐positive area and IHC staining. Scale bar = 200 µm. (M) WB analysis of F4/80, CD86, A2AR, SOCS1, p‐NF‐κB, NF‐κB, α‐SMA, and Col1a1 expression in the liver, normalized to β‐actin. (N) RT‐qPCR analysis for the expression of mRNA TNF‐α, IL‐6, and IL‐1β, as well as miR155 in the liver; β‐actin and U6 genes were used as the internal controls for mRNA and miRNA, respectively. All data were analyzed using unpaired Student's t‐test and expressed as mean ± SEM, *p‐value ˂ 0.05, **p‐value ≤ 0.01, N.S: *p*‐value ≥ 0.05, *n* ≥ 3.

Furthermore, FMT‐N‐administered mice exhibited higher levels of serum LPS, TNF‐α, and IL‐6 (except for IL‐1β) compared to mice in the FMT‐C group (Figure 4H), suggesting the activation of systemic inflammation. In terms of liver function, ALT and AST levels were significantly increased, whereas no significant differences were observed in the liver/body weight ratio (Figure 4I). Meanwhile, FMT‐N induced the infiltration of inflammatory cells and increased the PSR‐positive area in the liver (Figure 4K,L), indicating the pro‐inflammatory and pro‐fibrotic effects of H‐NPs‐induced gut microbiota. These effects were further supported by the upregulation of inflammatory cytokines TNF‐α and IL‐1β at the mRNA level (except for IL‐6) (Figure 4N) and elevated levels of the fibrosis‐related proteins α‐SMA and Col1a1 (Figure 4K–M). Moreover, H‐NPs‐induced macrophage recruitment and M1 polarization were reproduced by FMT‐N, as demonstrated by increased expression of F4/80 and CD86 (Figure 4K–M). Along with a decrease in inosine in serum and liver, FMT‐N also decreased the expression of A2AR (Figure 4J,M), suggesting that gut microbiota mediate H‐NPs‐induced decrease in liver inosine, which consequently reduces A2AR expression. In fact, A2AR has been reported as a crucial modulator of M1 macrophage polarization [34]. Additionally, compared to the FMT‐C group, FMT‐N elevated miR155 expression and the p‐NF‐κB/NF‐κB ratio, while reducing SOCS1 expression (Figure 4M,N), suggesting the activation of the miR155/SOCS1/NF‐κB pathway. These results indicate that gut microbiota mediate NPs‐induced polarization of M1 macrophages, which may be closely associated with A2AR and the miR155/SOCS1/NF‐κB pathway.

### 2.4 *B.p* Colonization Mitigated NPs‐Induced Impairment of Gut Homeostasis, Systemic Inflammation, and Hepatic Injury

Based on the analysis of microbial diversity and untargeted metabolomics, we found that probiotic *B.p* and its metabolite inosine were markedly decreased after H‐NPs exposure. Hence, we investigated whether *B.p* colonization could ameliorate H‐NPs‐induced damage of gut homeostasis (Figure 5A). Although *B.p* colonization failed to ameliorate H‐NPs‐induced slow rate of body weight gain (Figure 5B), it effectively mitigated the shortening of colonic length (Figure 5C). H‐NPs‐induced decrease in the number of AB^+^ and PAS^+^ goblet cells was also alleviated by *B.p* colonization (Figure 5D,E). Meanwhile, *B.p* partially reversed H‐NPs‐induced reduction and arrangement disorder of intestinal microvilli (Figure 5D). The significant decrease in the number of TUNEL‐positive cells demonstrated the anti‐apoptotic potential of *B.p* (Figure 5D,E). Moreover, *B.p* could protect against H‐NPs‐induced impairment of the intestinal barrier, as evidenced by the reversal of H‐NPs‐induced decreased expression of tight junction proteins ZO‐1, Occludin, and Claudin‐5 (Figure 5D–F). In addition, *B.p* colonization normalized H‐NPs‐induced high expression of inflammatory cytokines TNF‐α, IL‐6, and IL‐1β at the mRNA level (Figure 5G). These results suggest that *B.p* ameliorates NPs‐induced impairment of the intestinal barrier and colonic inflammation.

**FIGURE 5:**
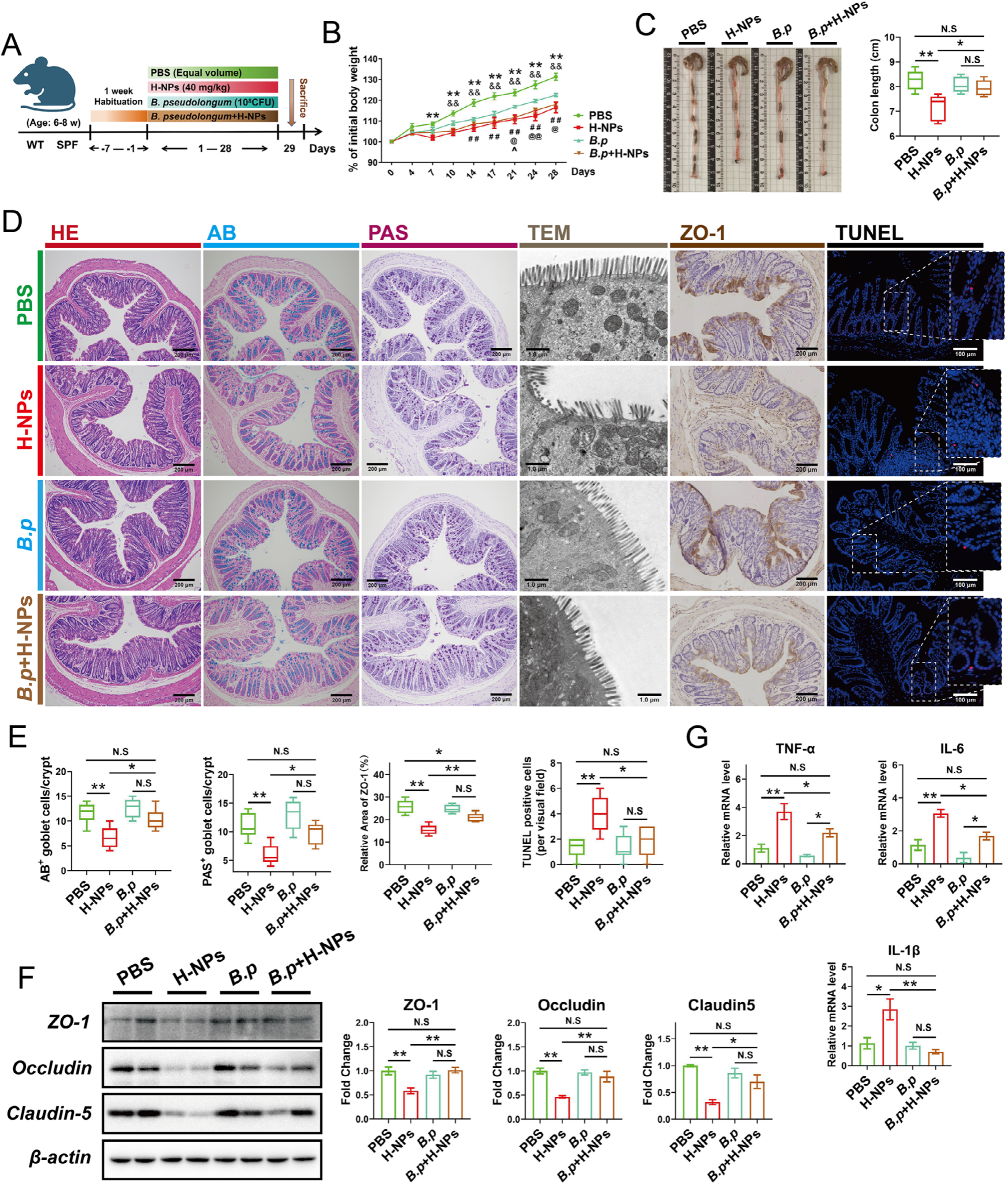
*B.p* colonization mitigated NPs‐induced impairment of the intestinal barrier and colonic inflammation. (A) Procedure for constructing the *B.p* colonization model in mice. (B) Changes in the percentage of initial body weight across the PBS, H‐NPs, *B.p*, and *B.p* + H‐NPs groups. Two‐way ANOVA was performed for statistical comparisons across the groups. **p‐value ≤ 0.01 (H‐NPs vs PBS); ^&&^ *p*‐value ≤ 0.01 (*B.p* + H‐NPs vs PBS); ^##^ *p*‐value ≤ 0.01 (*B.p* vs PBS); ^@^ *p*‐value ˂ 0.05, ^@@^ *p*‐value ≤ 0.01 (*B.p* vs H‐NPs); ^*p*‐value ˂ 0.05 (*B.p* + H‐NPs vs H‐NPs). (C) Representative images of the colon after PBS, H‐NPs, *B.p*, and *B.p* + H‐NPs administration and alteration in colonic length. (D, E) Representative images of HE, AB, PAS, and IHC staining for ZO‐1 (scale bar = 200 µm), TEM (scale bar = 1 µm), and TUNEL staining (scale bar = 100 µm) of the colon; the corresponding quantification of AB^+^ and PAS^+^ goblet cells per crypt, the relative area of ZO‐1, and the number of TUNEL‐positive cells per field. (F) WB analysis of ZO‐1, Occludin, and Claudin‐5 expression in the colon, normalized to β‐actin. (G) RT‐qPCR analysis for the relative expression of mRNA TNF‐α, IL‐6, and IL‐1β in the colon; β‐actin gene was used as the internal control. All data were analyzed using one‐way ANOVA with post hoc Tukey and expressed as mean ± SEM, *p‐value ˂ 0.05, **p‐value ≤ 0.01, N.S: *p*‐value ≥ 0.05, *n* ≥ 3.

Next, we explored the protective effects of *B.p* against H‐NPs‐induced systemic inflammation and hepatic injury. Effectively, H‐NPs‐induced upregulation of serum LPS, IL‐6, and IL‐1β (except for TNF‐α) was alleviated by *B.p* colonization (Figure 6A), indicating the amelioration of H‐NPs‐induced systemic inflammation. As shown in Figure 6B, *B.p* significantly mitigated H‐NPs‐induced elevation of liver/body weight ratio and serum ALT and AST levels, indicating the normalization of liver function. As a metabolite of *B.p*, H‐NPs‐induced low levels of inosine in serum and liver were partially restored by *B.p* colonization (Figure 6C). Meanwhile, H‐NPs‐induced infiltration of inflammatory cytokines and an increase in PSR‐positive area in the liver were repressed by *B.p* colonization (Figure 6D,E). Correspondingly, *B.p* effectively suppressed H‐NPs‐induced overexpression of fibrosis‐related proteins α‐SMA and Col1a1 (Figure 6D–F), as well as inflammatory cytokines TNF‐α and IL‐1β (except for IL‐6) (Figure 6G). These indicated that *B.p* could mitigate H‐NPs‐induced hepatic fibrosis and inflammation. Furthermore, the influence of *B.p* on H‐NPs‐induced polarization of M1 macrophages was evaluated. We found that H‐NPs‐induced increase in the expression of F4/80 and CD86 was ameliorated by *B.p* colonization (Figure 6D–F). These results suggest that *B.P* inhibits H‐NPs‐induced recruitment of macrophages and M1 polarization. Mechanically, *B.p* normalized H‐NPs‐induced decrease in A2AR (Figure 6F), which could be attributed to the reversal of low inosine level in the liver. Additionally, *B.p* colonization suppressed the miR155/SOCS1/NF‐κB pathway, a crucial regulatory signaling of M1 polarization, as demonstrated by the lower miR155 expression, the higher SOCS1, and the lower p‐NF‐κB/NF‐κB ratio in comparison with the H‐NPs group (Figure 6F,G). These findings illustrate that *B.p* may mitigate NPs‐induced hepatic injury by inhibiting M1 macrophage polarization.

**FIGURE 6:**
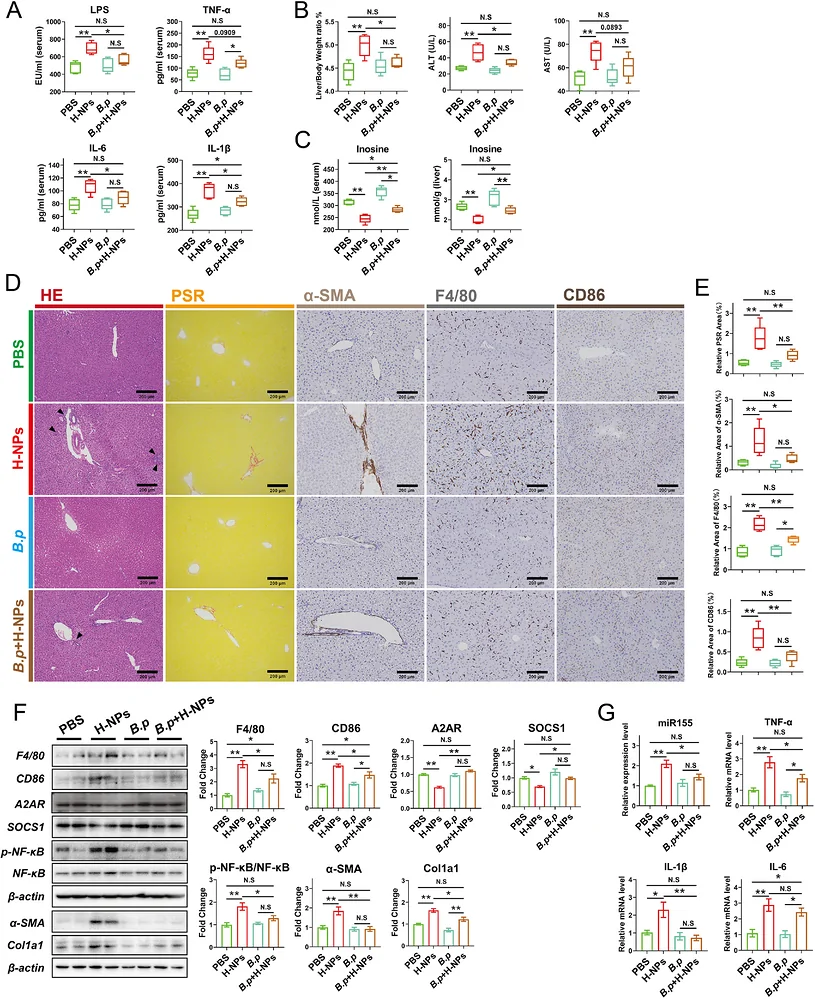
*B.p* colonization attenuated NPs‐induced systemic inflammation and hepatic injury. (A) Alterations in inflammatory cytokines: LPS, TNF‐α, IL‐6, and IL‐1β levels in the serum across the PBS, H‐NPs, *B.p*, and *B.p* + H‐NPs groups. (B) Alterations in liver/body weight ratio and liver function indexes: ALT and AST across the groups. (C) Alterations in inosine levels in the serum and liver across the groups. (D, E) Representative images of HE, PSR, and IHC staining for α‐SMA, F4/80, and CD86 in the liver, and the corresponding quantification of PSR‐positive area and IHC staining. Scale bar = 200 µm. (F) WB analysis of F4/80, CD86, A2AR, SOCS1, p‐NF‐κB, NF‐κB, α‐SMA, and Col1a1 expression in the liver, normalized to β‐actin. (G) RT‐qPCR analysis for the expression of mRNA TNF‐α, IL‐6, and IL‐1β, as well as miR155 in the liver; β‐actin and U6 genes were used as the internal controls of mRNA and miRNA, respectively. All data were analyzed using one‐way ANOVA with post hoc Tukey and expressed as mean ± SEM, *p‐value ˂ 0.05, **p‐value ≤ 0.01, N.S: *p*‐value ≥ 0.05, *n* ≥ 3.

### 2.5 Inosine Mediated the Protection of *B.p* against NPs‐Induced Hepatic Injury by Inhibiting the Polarization of M1 Macrophages

We discovered that *B.p* colonization could ameliorate H‐NPs‐induced hepatic injury, accompanied by the elevation of its metabolite inosine in serum and liver. Based on this, we hypothesized that inosine mediates the protective effects of *B.p*. Before examining the protective effects of inosine, we investigated whether *B.p* exerted its ameliorative effects by altering the physicochemical properties of NPs through its metabolite inosine. As shown in Figure 7A–C, inosine did not significantly alter the surface morphological structure, particle size, or surface functional groups of NPs at various co‐incubation temperatures. Meanwhile, NPs also did not exhibit effective adsorption of inosine (Figure 7D). As observed in inosine co‐incubation, the surface morphological structure, particle size, and surface functional groups of NPs were not altered after *B.p* supernatant incubation (Figure 7A–C). Interestingly, both Inosine and *B.p* supernatant reduced the zeta potential of NPs (Figure 7E). These results suggest that *B.p* and its metabolite inosine have a limited impact on the physicochemical properties of NPs.

**FIGURE 7:**
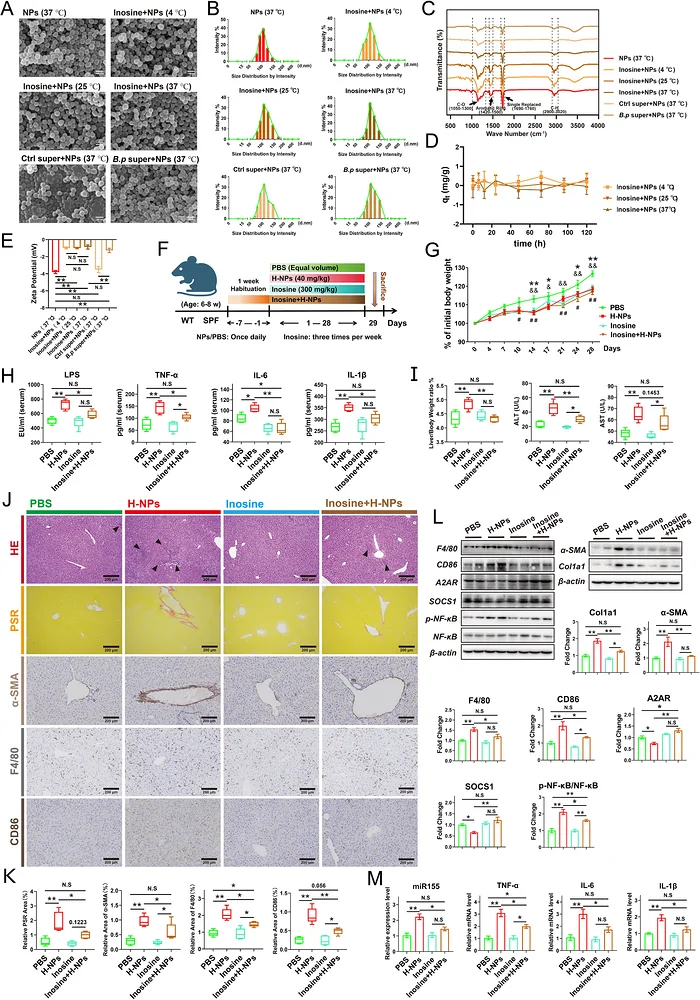
Inosine ameliorated NPs‐induced hepatic injury by repressing the polarization of M1 macrophages. (A, B) Morphological characteristics of NPs and the distribution of particle size across the NPs (37°C), Inosine + NPs (4°C), Inosine + NPs (25°C), Inosine + NPs (37°C), Ctrl super + NPs (37°C), and *B.p* super + NPs (37°C) groups (SEM HV = 10.0 kV, scale bar = 100 nm). (C) Alterations in the Fourier infrared spectrum of NPs across groups. (D) Kinetic curve of inosine adsorption on NPs. (E) Changes in the Zeta potential of NPs across groups. (F) Procedure for constructing the inosine supplementation model in mice. (G) Alterations in the percentage of initial body weight across PBS, H‐NPs, Inosine, and Inosine + H‐NPs groups. Two‐way ANOVA was performed for statistical comparisons across the groups. *p‐value ˂ 0.05, **p‐value ≤ 0.01 (H‐NPs vs. PBS); ^&^ *p*‐value ˂ 0.05, ^&&^ *p*‐value ≤ 0.01 (Inosine + H‐NPs vs PBS); ^#^ *p*‐value ˂ 0.05, ^##^ *p*‐value ≤ 0.01 (Inosine vs PBS). (H) Alterations in inflammatory cytokines: LPS, TNF‐α, IL‐6, and IL‐1β levels in the serum across the groups. (I) Alterations in liver/body weight ratio and liver function indexes: ALT and AST across the groups. (J, K) Representative images of HE, PSR, and IHC staining for α‐SMA, F4/80, and CD86 in the liver, and the corresponding quantification of PSR‐positive area and IHC staining. Scale bar = 200 µm. (L) WB analysis of F4/80, CD86, A2AR, SOCS1, p‐NF‐κB, NF‐κB, α‐SMA, and Col1a1 expression in the liver, normalized to β‐actin. (M) RT‐qPCR analysis for the expression of mRNA TNF‐α, IL‐6, and IL‐1β, as well as miR155 in the liver; β‐actin and U6 genes were used as the internal controls for mRNA and miRNA, respectively. All data were analyzed using one‐way ANOVA with post hoc Tukey and expressed as mean ± SEM, *p‐value ˂ 0.05, **p‐value ≤ 0.01, N.S: *p*‐value ≥ 0.05, *n* ≥ 3.

Next, a dosage of 300 mg kg^−1^ inosine was administered orally to mice three times per week to evaluate its protective effects (Figure 7F). This dosing regimen has been previously documented to replicate the protective effects of *B.p* [35, 36]. Further, we determined the concentrations of inosine in both serum and liver after *B.p* colonization and inosine supplementation. Our results suggested that there were no significant differences in serum and liver levels of inosine between the *B.p* and Inosine groups. Similarly, no significant differences were observed in inosine levels between the *B.p* + H‐NPs and Inosine + H‐NPs groups (Figure S4A). Therefore, 300 mg kg^−1^ inosine can reflect the physiological increase in inosine induced by *B.p* colonization. Similar to *B.p* colonization, inosine failed to alleviate H‐NPs‐induced slow rate of body weight gain (Figure 7G). Effectively, inosine ameliorated H‐NPs‐induced systemic inflammation by inhibiting the elevation of serum LPS, TNF‐α, IL‐6, and IL‐1β (Figure 7H). The protective effects of *B.p* against H‐NPs‐induced impairment of liver function were replicated by inosine supplementation, as demonstrated by the reversal of elevated liver/body weight ratio, ALT, and AST levels (no significant difference) (Figure 7I). Moreover, inosine alleviated H‐NPs‐induced infiltration of inflammatory cells and the increase in PSR‐positive area in the liver (Figure 7J,K). The anti‐inflammatory and anti‐fibrotic effects of inosine were further confirmed at the molecular level. H‐NPs‐induced overexpression of fibrosis‐related proteins α‐SMA and Col1a1 was mitigated by inosine supplementation (Figure 7J–L). Furthermore, inosine alleviated H‐NPs‐induced elevation of hepatic inflammation cytokines TNF‐α, IL‐6, and IL‐1β at the mRNA level (Figure 7M). In addition, inosine reproduced *B.p*‐induced repression of H‐NPs‐induced macrophage recruitment and M1 polarization, as evidenced by the inhibition of F4/80 and CD86 expression (Figure 7J–L). As observed in the *B.p* colonization experiment, inosine also overexpressed its target protein A2AR and suppressed the miR155/SOCS1/NF‐κB pathway, as demonstrated by normalized miR155 and SOCS1 expression and decreased p‐NF‐κB/NF‐κB ratio (Figure 7L,M). To directly explore the role of macrophages in NPs‐induced hepatic injury and the ameliorative effect of inosine, we established a co‐culture system of AML12 and RAW264.7 cell lines. We found that co‐culture with NPs‐administered RAW264.7 cells significantly increased the apoptosis ratio of AML12 cells and elevated the supernatant levels of ALT and AST, whereas RAW264.7 cells treated with inosine did not display significant injury to AML12 cells regardless of NPs administration (Figure 8A,B). These results suggest that inosine mediates the protective effects of *B.p* against H‐NPs‐induced hepatic injury by inhibiting M1 macrophage polarization.

**FIGURE 8:**
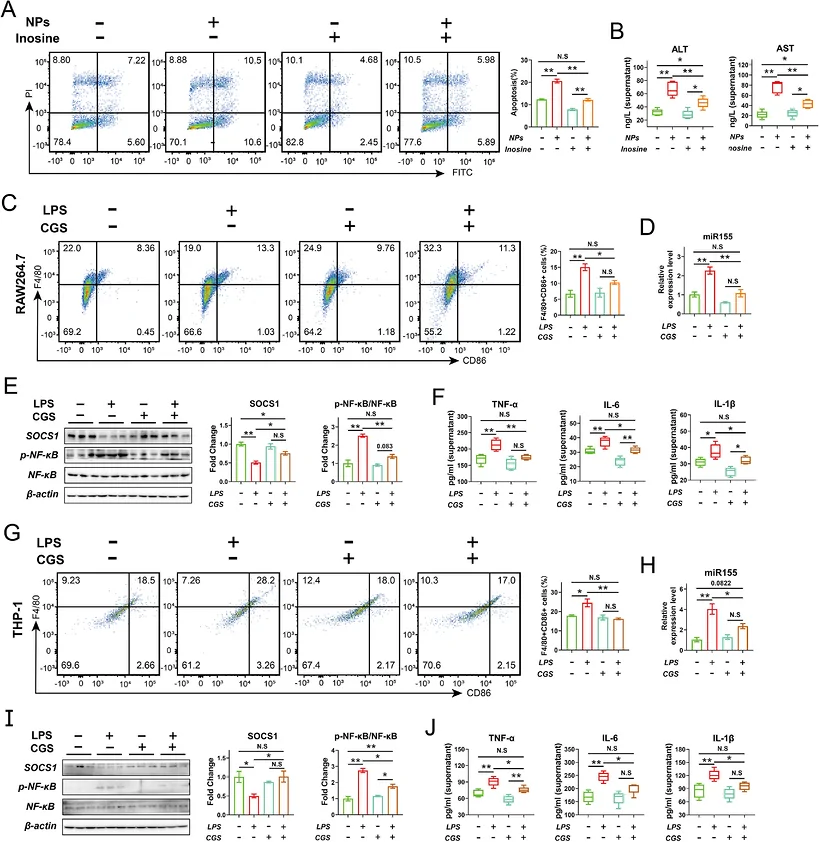
CGS21680 repressed LPS‐induced activation of the miR155/SOCS1/NF‐κB pathway and inhibited M1 macrophage polarization in vitro. (A) Representative analysis of the percentage of FITC^+^ PI^+^ AML12 cells detected using flow cytometry in the DMSO, NPs, Inosine, and Inosine + NPs groups. (B) Alterations in liver function indexes: ALT and AST across the groups. (C) Representative analysis of the percentage of F4/80^+^ CD86^+^ RAW264.7 cells detected using flow cytometry in the DMSO, LPS, CGS21680, and CGS21680 + LPS groups. (D) RT‐qPCR analysis for the expression of miR155 in RAW264.7 cells; U6 was used as an internal control. (E) WB analysis of SOCS1, p‐NF‐κB, and NF‐κB expression in RAW264.7 cells, normalized to β‐actin. (F) Concentrations of the inflammatory cytokines TNF‐α, IL‐6, and IL‐1β in culture supernatants across the groups. (G) Representative analysis of the percentage of F4/80^+^ CD86^+^ THP‐1 cells detected using flow cytometry in the DMSO, LPS, CGS21680, and CGS21680 + LPS groups. (H) RT‐qPCR analysis for the expression of miR155 in THP‐1 cells; U6 was used as an internal control. (I) WB analysis of SOCS1, p‐NF‐κB, and NF‐κB expression in THP‐1 cells, normalized to β‐actin. (J) Concentrations of the inflammatory cytokines TNF‐α, IL‐6, and IL‐1β in culture supernatants across the groups. All data were analyzed using one‐way ANOVA with post hoc Tukey and expressed as mean ± SEM, *p‐value ˂ 0.05, **p‐value ≤ 0.01, N.S: *p‐*value ≥ 0.05, *n* ≥ 3.

To evaluate the equivalence of the protective effects between *B.p* colonization and inosine supplementation, we performed comparisons of indexes related to systemic inflammation and hepatic injury. In terms of systemic inflammation, both *B.p* and inosine alleviated H‐NPs‐induced increase in serum levels of LPS, TNF‐α, IL‐6, and IL‐1β with no statistical differences observed between the *B.p* + H‐NPs and Inosine + H‐NPs groups (Figure S5A). Similar phenomena were observed in hepatic injury‐related indexes, including hepatic function indexes ALT and AST (although no statistical difference), PSR‐ and α‐SMA‐positive area (associated with fibrosis), and F4/80‐ and CD86‐positive area (associated with M1 macrophage polarization) (Figure S5B–D). These suggest that a dosage of 300 mg kg^−1^ inosine confers a protective effect equivalent to that of *B.p* colonization.

### 2.6 A2AR Mediated the Suppressive Effect of Inosine on NPs‐Induced Polarization of M1 Macrophages by Inhibiting the miR155/SOCS1/NF‐κB Pathway

To further elucidate whether inosine inhibited the miR155/SOCS1/NF‐κB pathway by targeting A2AR, the A2AR agonist CGS21680 was utilized to treat LPS‐administered murine‐derived macrophage cell line RAW264.7. LPS significantly increased the ratio of F4/80‐positive CD86‐positive (F4/80^+^ CD86^+^) RAW264.7 cells, which was effectively repressed by CGS21680 treatment (Figure 8C). These results suggest inhibition of LPS‐induced M1 macrophage polarization. Accompanied by decreased levels of TNF‐α, IL‐6, and IL‐1β, CGS21680 reversed LPS‐induced upregulation of miR155 and p‐NF‐κB/NF‐κB ratio, as well as the downregulation of SOCS1 (Figure 8D–F), indicating inhibition of the miR155/SOCS1/NF‐κB pathway. To further validate these results, the human‐derived THP‐1 cell line was used to establish the CGS21680 intervention model. It was observed that CGS21680 also effectively repressed LPS‐induced increase in the F4/80^+^ CD86^+^ ratio in THP‐1 cells (Figure 8G). Concurrently, LPS‐induced elevation of miR155 and p‐NF‐κB/NF‐κB ratio, as well as the decrease of SOCS1, were mitigated by CGS21680 treatment (Figure 8H,I). Additionally, CGS21680 normalized LPS‐induced overexpression of the inflammatory cytokines TNF‐α, IL‐6, and IL‐1β (Figure 8J). These evidence indicate that A2AR has the capacity to inhibit the miR155/SOCS1/NF‐κB pathway and repress M1 macrophage polarization.

To elucidate the role of miR155 in NPs‐induced M1 macrophage polarization, we introduced miR155 knockout mice (miR155^−/−^) to establish the H‐NPs exposure model (Figure 9A,B). miR155 knockout significantly repressed H‐NPs‐induced elevation in serum LPS, TNF‐α, IL‐6, and IL‐1β, indicating the amelioration of systemic inflammation (Figure 9C). Moreover, we assessed the impact of miR155 knockout on inosine concentrations in both serum and liver. Compared to mice in the wild‐type (WT)‐PBS group, the inosine levels in serum and liver were not significantly altered after miR155 knockout; similarly, miR155 knockout also did not alleviate H‐NPs‐induced reduction of inosine in these tissues (Figure S6A). In terms of liver function, the H‐NPs‐induced increase in serum ALT and AST levels was normalized by miR155 knockout (Figure 9D), suggesting the mitigation of liver function impairment. Meanwhile, H‐NPs‐administered miR155^−/−^ mice exhibited milder inflammation and fibrosis in the liver compared to the WT mice. The anti‐inflammatory effects of miR155 knockout were reflected by reduced infiltration of inflammatory cells and lower mRNA levels of TNF‐α, IL‐6, and IL‐1β (Figure 9E,H), while its anti‐fibrotic effects were demonstrated by the reduced PSR‐positive area and normalization of fibrosis‐related proteins α‐SMA and Col1a1 in the liver (Figure 9E–G). In addition, H‐NPs‐induced macrophage recruitment and M1 polarization were reversed by miR155 knockout, as evidenced by the lower expression of F4/80 and CD86 (Figure 9E–G). As the downstream of miR155, the SOCS1/NF‐κB pathway was inhibited by miR155 knockout, regardless of H‐NPs exposure, as evidenced by normalization of SOCS1 and p‐NF‐κB/NF‐κB ratio (Figure 9F,G). However, miR155 knockout did not affect the inhibition of H‐NPs on A2AR (Figure 9F,G). In vitro, CGS21680 overexpressed A2AR and alleviated LPS‐induced increase in the ratio of F4/80^+^ CD86^+^ RAW264.7 cells (Figure S7A). CGS21680 also inhibited the miR155/SOCS1/NF‐κB pathway, along with a reduction of the inflammatory cytokines TNF‐α, IL‐6, and IL‐1β (Figure S7B–D). Meanwhile, the miR155 inhibitor also led to a reduction in the ratio of F4/80^+^ CD86^+^ RAW264.7 cells and repression of the downstream SOCS1/NF‐κB pathway, accompanied by a decrease in inflammatory cytokines. However, the miR155 inhibitor did not affect LPS‐induced low expression of A2AR (Figure S7A–D), indicating that A2AR is located upstream of miR155. These results suggest that A2AR may mitigate NPs‐induced polarization of M1 macrophages by inhibiting the miR155/SOCS1/NF‐κB pathway.

**FIGURE 9:**
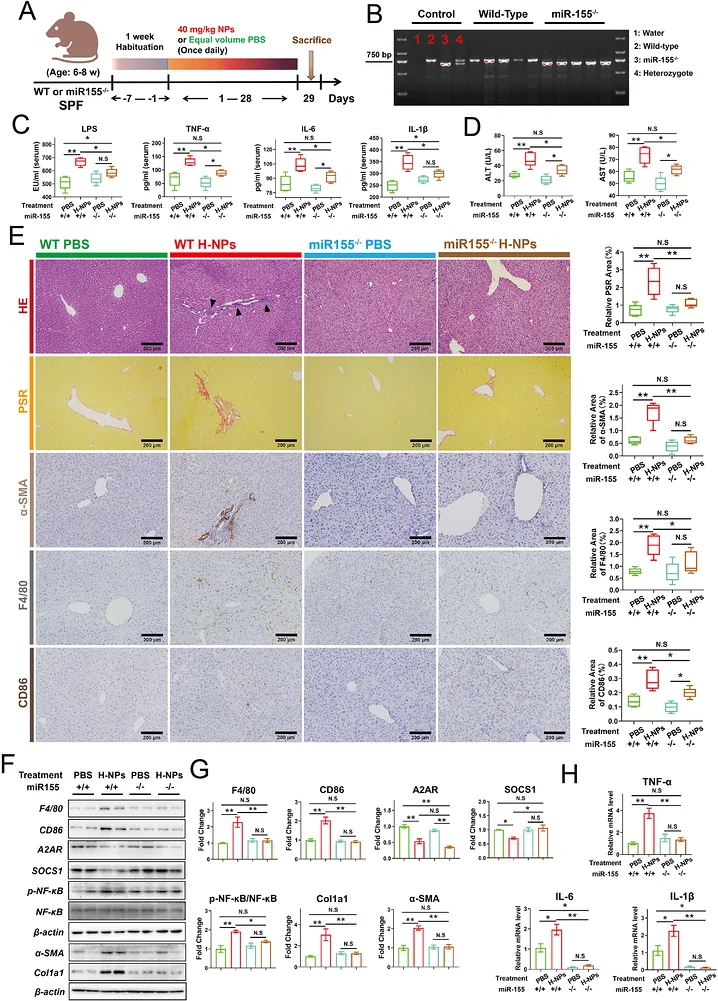
MiR155 knockout mitigated NPs‐induced hepatic injury by suppressing the polarization of M1 macrophages. (A) Procedure for constructing the H‐NPs exposure model in WT and miR155 knockout (miR155^−/−^) mice. (B) Genotype identification of miR155^−/−^ mice with agarose gel electrophoresis. (C) Alterations in inflammatory cytokines: LPS, TNF‐α, IL‐6, and IL‐1β levels in the serum across the WT‐PBS, WT‐H‐NPs, miR155^−/−^‐PBS, and miR155^−/−^‐H‐NPs groups. (D) Alterations in liver function indexes: ALT and AST across the groups. (E) Representative images of HE, PSR, and IHC staining for α‐SMA, F4/80, and CD86 in the liver, and the corresponding quantification of PSR‐positive area and IHC staining. Scale bar = 200 µm. (F, G) WB analysis of F4/80, CD86, A2AR, SOCS1, p‐NF‐κB, NF‐κB, α‐SMA, and Col1a1 expression in the liver, normalized to β‐actin. (H) RT‐qPCR analysis for the expression of mRNA TNF‐α, IL‐6, and IL‐1β in the liver; β‐actin gene was used as the internal control. All data were analyzed using one‐way ANOVA with post hoc Tukey and expressed as mean ± SEM, *p‐value ˂ 0.05, **p‐value ≤ 0.01, N.S: *p‐*value ≥ 0.05, *n* ≥ 3.

Finally, we investigated the potential synergistic effect of inosine and miR155 inhibitor on anti‐fibrosis. The results demonstrated that both inosine and miR155 inhibitor effectively repressed TGF‐β1‐induced upregulation of fibrosis‐related proteins Col1a1 and α‐SMA in murine hepatic stellate cells (mHSCs). However, the combined use of inosine and miR155 inhibitor did not result in a further reduction of Col1a1 and α‐SMA expression compared to the use of either agent alone (Figure S7E). These indicate that inosine and miR155 inhibitor do not exhibit a synergistic effect on anti‐fibrosis.

## 3 Discussion

The extensive use of plastic products has caused serious environmental pollution globally [1]. Nanoplastics (NPs), derived from plastic products, have emerged as a health risk to humans due to their biotoxicity [37]. As the major site for drug catabolism, the liver is recognized as a crucial target organ for NPs toxicity [5]. Consequently, it is necessary to elucidate the mechanisms of NPs‐induced hepatic injury and investigate effective intervention strategies. In this study, NPs lowered the abundance of probiotic *B.p* and the concentration of its metabolite inosine. FMT experiment indicated that gut microbiota mediated NPs‐induced impairment of gut homeostasis and contributed to hepatic injury by promoting M1 macrophage polarization. Moreover, *B.p* colonization mitigated NPs‐induced gut homeostasis impairment and attenuated hepatic injury. Inosine supplementation reproduced the protective effects of *B.p* against NPs‐induced hepatic injury. We confirmed that *B.p‐*derived inosine targeted A2AR and subsequently repressed the miR155/SOCS1/NF‐κB pathway, thereby inhibiting NPs‐induced M1 macrophage polarization. Our findings reveal that gut microbiota participate in NPs‐induced hepatic injury by modulating M1 polarization, whereas *B.p* colonization may serve as a potential microbial intervention strategy.

There is accumulating evidence that gut microbiota are strictly associated with the occurrence and development of multiple liver diseases [12, 38]. In this study, we also observed the disturbance of gut microbiota after NPs exposure, notably a significant reduction in the abundance of probiotic *B.p*. Previous studies have demonstrated that *B.p* exhibits effective protection against multiple colonic and liver diseases. *B.p* has been shown to slow the progression of NAFLD‐associated hepatocellular carcinoma by modulating the G protein‐coupled receptor 43 to inhibit the IL‐6/JAK1/STAT3 pathway [39]. Furthermore, it has been reported that *B.p* mediated the amelioration of Indole‐3‐acetic acid on DSS‐induced colitis by promoting the production of R‐equol [40]. *Akkermansia muciniphila* was another significantly reduced probiotic after NPs administration, which was also observed to decrease with MPs exposure in our previous study [9]. Liu et al. found that by modulating the synthesis of retinoic acid, *Akkermansia muciniphila* ameliorated DSS‐induced colitis [41]. Additionally, *Akkermansia muciniphila* also protected against nonalcoholic steatohepatitis by modulating the IL‐17‐mediated macrophage polarization [42]. Moreover, NPs exposure resulted in a reduced abundance of probiotics *Lactobacillus murinus*. Recently, a study has reported that *Lactobacillus murinus* alleviates ischemia/reperfusion‐induced intestinal injury by promoting the release of IL‐10 from M2 macrophages [43]. *Lactobacillus murinus* also mediated the protection of time‐restricted feeding against septic liver injury through its metabolite hydroxybutyrate [44]. These evidence suggest that a reduction in probiotics may be involved in NPs‐induced gut homeostasis impairment and hepatic injury.

Gut metabolites serve as an important link between the gut microbiota and the liver [11]. Here, fecal metabolomics revealed that NPs resulted in a significant decrease in inosine and its derivatives 1‐methylhypoxanthine and 7‐methylhypoxanthine, with nucleotide metabolism identified as the most enriched pathway. In fact, inosine is an important purine nucleotide of host‐microbial co‐metabolism with various biological activities, including regulating immunity and cellular energy metabolism [45, 46]. Recently, inosine has been identified as the major metabolite of *B.p*, which mediated *B.p*‐induced enhancement of immune checkpoint blockade [45]. Depletion of *B.p* and its metabolite inosine mediated the promoting effect of inulin on the development and progression of colorectal cancer [47]. These findings imply that microbial dysbiosis, especially a decrease in *B.p* and its metabolite inosine, contributes to NPs‐related impairment of gut homeostasis and hepatic injury. Indeed, NPs‐induced deficit in gut homeostasis and liver function was reproduced by fecal microbiota transplantation. Further, we investigated the potential protection of *B.p* and its metabolite inosine. We found that *B.p* colonization effectively ameliorated NPs‐induced hepatic inflammation and fibrosis, and its protective effects were reproduced by inosine supplementation. As the key metabolite of *B.p*, inosine has presented various biological activities, including modulating intestinal innate immunity and maintaining liver function. By elevating microbiota‐derived inosine levels, barley leaf effectively mitigated the experimental colitis by stimulating the A2AR/PPARγ pathway [48]. In addition, inosine repressed NAFLD‐induced hepatic inflammation and pyroptosis by enhancing fatty acid β‐oxidation [49]. These findings indicate that the protective effects of *B.p* are attributed to its metabolite inosine.

Previous studies have demonstrated that macrophages play a crucial role in interactions between the gut microbiota and the liver [50, 51]. In this study, NPs promoted the polarization of M1 macrophages in the liver by activating the miR155/SOCS1/NF‐κB pathway, which was replicated by FMT. This suggests that gut microbiota mediate NPs‐induced M1 macrophage polarization. Jiang et al. reported that inosine could reduce LPS‐induced release of inflammatory cytokines from M1 macrophages by inhibiting HIF‐1α through interaction with prolyl hydroxylase [52]. By promoting metabolic reprogramming of purine, L‐arginine enhanced inosine levels and subsequently inhibited osteoclastogenesis (a subtype of macrophages), thereby improving arthritis and inflammatory bone loss [53]. These findings indicate that gut microbiota‐induced polarization of M1 macrophages may be attributed to a decrease in inosine. Moreover, along with the upregulation of A2AR, both *B.p* and its metabolite inosine repressed NPs‐induced M1 macrophage polarization by inhibiting the miR155/SOCS1/NF‐κB pathway. A2AR is a G protein‐coupled receptor with high affinity for adenosine and is highly expressed in immune cells, including macrophages [54]. In fact, A2AR has exhibited the protective effects of self‐tissues from autoimmune responses by influencing the function of macrophages. Activation of A2AR attenuated progressive nephropathy‐induced release of inflammatory cytokines and fibrosis, whereas this effect was eliminated by A2AR knockout [55]. Meanwhile, A2AR could promote the expression of anti‐inflammatory cytokine IL‐10 in macrophages, thereby protecting against sepsis‐induced “inflammatory storm” [34]. Modulation of macrophages by A2AR was also observed in this study, as it effectively repressed NPs‐induced polarization of M1 macrophages in the liver by inhibiting the miR155/SOCS1/NF‐κB pathway.

Although we have confirmed that *B.p*‐derived inosine protects against NPs‐induced hepatic injury, the potential roles of other differential metabolites should not be overlooked. Paricalcitol, which was downregulated following NPs exposure, has been reported to mitigate NAFLD‐related oxidative stress and inflammation [56]. By promoting M2 macrophage polarization, paricalcitol alleviated diabetic nephropathy‐related inflammation [57]. Moreover, an increase in tryptophol, a secondary metabolite of *Candida albicans*, was observed following NPs administration. In vitro, tryptophol has exhibited obvious hepatocyte toxicity [58]. Furthermore, NPs significantly reduced the fecal level of indole‐3‐carboxylic acid. Li et al. demonstrated that *Bifidobacterium*‐derived indole‐3‐carboxylic acid mediated the protective effects of magnesium against acetaminophen‐induced hepatic injury by inhibiting oxidative stress [51]. Notably, there was a significant elevation of Sapidolide A after NPs exposure. It has been reported that Sapidolide A could ameliorate acetaminophen‐induced hepatic injury by repressing the activation of NLRP in macrophages [59]. This phenomenon implies that Sapidolide A may have a contrasting role in NPs‐induced hepatic injury. These findings suggest that, apart from inosine, some other metabolites may also serve as potential therapeutic candidates for NPs‐induced hepatic injury.

However, we have to admit that our research still has some shortcomings. Firstly, the study utilized a singular and standardized type and size of plastic, which cannot accurately represent real‐world exposure conditions. Secondly, we only focused on the toxic mechanisms of polystyrene plastic, and whether other plastic types have similar mechanisms is still unclear. Thirdly, this study did not investigate the impact of the physicochemical properties of plastic on its toxicity. The direct toxic effects of NPs on gut microbiota also need to be evaluated. Furthermore, as the study was conducted in mice, additional research is necessary to determine the applicability of the findings to humans. Additionally, the safety of long‐term use of *B.p* and inosine also requires further assessment. Lastly, the detailed mechanisms underlying the modulation of A2AR to the miR155 pathway have yet to be clarified thoroughly.

## 4 Conclusion

In summary, our work demonstrated that gut microbiota mediated NPs‐induced damage of gut homeostasis and hepatic injury. Meanwhile, reversing the decrease in *B.p* ameliorated NPs‐induced damage of gut homeostasis and hepatic injury. In addition, the protective effects of *B.p* on the liver were replicated by the supplementation of its metabolite inosine. Mechanically, inosine repressed NPs‐induced polarization of M1 macrophages by inhibiting the miR155/SOCS1/NF‐κB pathway via targeting A2AR. Altogether, this study further elucidated NPs‐induced hepatic injury from the perspective of gut microbiota and proposed a promising treatment strategy.

## 5 Methods

### 5.1 Animals and Reagents

Male C57BL/6 mice (6–8 weeks old, average weight of 22–24 g) were used in this study, which were obtained from the Laboratory Animal Center of Southern Medical University. During the animal experiments, mice were maintained under standard housing conditions with ad lib feeding (12/12 h light/dark cycle, 23°C, 55% humidity). All animal operations were conducted in strict accordance with the guidelines for animal care and use of Southern Medical University and approved by the Ethics Committee of Nanfang Hospital, Southern Medical University (IACUC‐LAC‐20230724‐003).

Polystyrene NPs (Cat#: 65568898989, particle size: 100 nm, over 98% purity) and polystyrene MPs (Cat#: PS12345953, particle size: 5 µm, over 98% purity) were purchased from Tesulang Chemical Materials Co., Ltd. (Dongguan, China). Antibiotic cocktail solutions were prepared using PBS containing 10 mg mL^−1^ vancomycin (Cat#: V301569, Aladdin, Shanghai, China), 20 mg mL^−1^ neomycin sulfate (Cat#: N109017, Aladdin), 20 mg mL^−1^ metronidazole (Cat#: M109874, Aladdin), and 20 mg mL^−1^ ampicillin (Cat#: A102048, Aladdin). Inosine was purchased from Sigma‐Aldrich Co., Ltd. (over 99% purity, Cat#: I4125, MO, USA). CGS21680 was obtained from Macklin Biochemical Co., Ltd. (Cat#: C884032, Shanghai, China). The miR155 inhibitor was purchased from RIBOBIO Co., Ltd. (Cat#: miR2CM001, Guangzhou, China). TGF‐β1 was obtained from MREDA Co., Ltd. (Cat#: M188199, Beijing, China). Lipopolysaccharide (LPS) was obtained from ACMEC Biochemical Co., Ltd. (Cat#: LPS, Shanghai, China).

### 5.2 Animal Treatments

Before experiments, all mice were acclimatized to the experimental environment for one week. According to the intended experimental design, the following six animal experiments were conducted.

Experiment for NPs or MPs exposure: NPs and MPs were prepared as a suspension in PBS at a concentration of 4 mg mL^−1^. Based on experimental design, mice were allocated into four groups: PBS, low‐dose NPs (L‐NPs, 20 mg kg^−1^), high‐dose NPs (H‐NPs, 40 mg kg^−1^), and high‐dose MPs groups (H‐MPs, 40 mg kg^−1^) groups (10 per group). The NPs and MPs were administered to mice via gavage once daily for 28 d [9, 14]. Mice in the PBS group were administered an equal volume of PBS and served as controls. It has been reported that human exposure to MPs or NPs was approximately 0.1–5 g per week [3]. According to the equivalency conversion in mice, the exposure dose adopted in this study was within the daily exposure range (2.16–108.4 mg kg^−1^).

Experiment for fluorescence imaging in vivo: After 12 h of fasting and water deprivation, 15 mice were randomly divided into the PBS, NPs, and MPs groups. Mice in the NPs and MPs groups received a single oral dose of fluorescently labeled NPs and MPs at a concentration of 40 mg kg^−1^, respectively. Mice in the PBS group were orally administered with an equal volume of PBS. 24 h after gavage, the tissue distribution of NPs and MPs in mice was assessed using an In‐Vivo Multispectral (MS) FX PRO system with excitation/emission wavelengths of 532 nm/585 nm.

Experiment for fecal microbiota transplantation (FMT): Feces for FMT were collected from donor mice (pretreated with PBS or H‐NPs for 28 d) using sterile metabolic cages. Feces were thoroughly mixed with sterile PBS at a ratio of 1 g/10 mL, and the obtained fecal suspensions were centrifuged to separate the bacterial solution (800 rpm, 3 min, 4°C). The recipient mice were orally pretreated with an antibiotic cocktail (10 mL kg^−1^) once daily for 1 week and randomly grouped into the FMT‐C and FMT‐N groups. Mice in the FMT‐C and FMT‐N groups were administered with bacterial solutions (10 mL kg^−1^ body weight) from PBS‐ and H‐NPs‐treated donors via oral gavage (once daily for 28 consecutive days), respectively (*n* = 10 per group) [33, 38].

Experiment for *B.p* colonization: Mice were randomly grouped into four groups: PBS, H‐NPs, *B.p*, and *B.p* + H‐NPs groups (*n* = 10 per group). Mice in the PBS and H‐NPs groups were orally administered with PBS or NPs as previously described. As well, mice in the *B.p* group were orally administered 200 µL *B.p* suspension (1 × 10^9^ CFU bacterium) once daily for a continuous 28 d, while those in the *B.p* + H‐NPs group received both *B.p* and NPs.

Experiment for the supplementation of *B.p* metabolite inosine: Mice were randomized into PBS, H‐NPs, Inosine, and Inosine + H‐NPs groups (*n* = 10 per group). Mice in the PBS and H‐NPs groups were treated as previously described. To evaluate the protection of inosine, mice were concurrently administered 300 mg kg^−1^ inosine (three times per week) and 40 mg kg^−1^ NPs for 28 d (once daily) by gavage. The Inosine group mice were orally administered inosine alone. The dosage of inosine has been previously documented to replicate the protective effects of *B.p* [35, 36].

Experiment for NPs exposure on miR155 knockout (miR155^−/−^) mice: According to genotype, wild‐type (WT), and miR155^−/−^ mice (obtained from Suzhou Saiye Biotechnology Co., Ltd., Guangzhou, China) were randomized into WT‐PBS, WT‐H‐NPs, miR155^−/−^‐PBS, and miR155^−/−^‐H‐NPs groups (*n* = 10 per group) and then orally administered PBS or H‐NPs as previously described.

### 5.3 Cell Culture and Treatment

Experiment for CGS21680 treatment: The murine‐derived macrophage RAW264.7 cell line (RRID: CVCL_0493) was acquired from iCell Biotechnology Co., Ltd. Mycoplasma testing was conducted regularly using a GMyc‐PCR Mycoplasma Test Kit (Cat#: 40601ES10, Yeasen, Shanghai, China) to ensure no mycoplasma contamination. RAW264.7 cells were cultured in RPMI 1640 medium supplemented with 10% fetal bovine serum and 1% penicillin–streptomycin double antibody, at a density of 1 × 10^6^ cells cm^−2^ for 24 h. The cells were maintained at 37°C with 5% CO_2_, 21% O_2_, and 74% N_2_. RAW264.7 cells in the LPS group were stimulated using LPS (1 µg mL^−1^) to induce M1 polarized macrophages for 24 h; RAW264.7 cells in the CGS group were incubated with CGS21680 (1 µm); CGS + LPS group cells received concurrent treatment with both CGS21680 and LPS. DMSO was used as a negative control and solvent for LPS and CGS21680. Finally, culture supernatants and cells were collected for further analysis. To test the generalizability of our findings across species, the human‐derived THP‐1 cell line (RRID: CVCL_0006) was sourced from Shanghai Zhong Qiao Xin Zhou Biotechnology Co., Ltd. The incubation conditions, grouping, and interventions of the THP‐1 cells were the same as described above.

Experiment for co‐culture model of hepatocytes and macrophages: The murine‐derived hepatocyte cell line AML12 (RRID: CVCL_0140) and RAW264.7 (RRID: CVCL_0493) were purchased from iCell Biotechnology Co., Ltd. Mycoplasma contamination testing was performed regularly. Raw264.7 cells were divided into the DMSO, NPs, Inosine, and Inosine + NPs groups. The cells in the NPs group were exposed to 500 µg mL^−1^ NPs for 48 h. The Inosine group received a treatment of 1 mm inosine for the same period. The Inosine + NPs group cells were subjected to both treatments concurrently for 48 h. DMSO served as the blank control group. After induction, the separation of Raw264.7 cells from the culture medium was performed. Then, Raw264.7 and AML12 cells were co‐cultured using 12‐well Corning Transwell plates equipped with a permeable cross‐hole insert (aperture 0.4 µm). Raw264.7 cells were cultured in the upper chamber at a density of 4 × 10^5^ cells cm^−2^, while AML12 cells were cultured in the lower chamber at the same density. After co‐culture for 24 h, AML12 cells and the culture supernatant were collected for further analysis.

Experiment for CGS21680 and miR155 inhibitor treatment: RAW264.7 cells were assigned to four groups: LPS, CGS + LPS, miR155 inhibitor + LPS, and CGS + miR155 inhibitor + LPS groups. Briefly, RAW264.7 cells in the LPS group were treated with 1 µg mL^−1^ LPS for 24 h; RAW264.7 cells in the LPS + CGS group were concurrently treated with LPS and 1 µm CGS21680; in the miR155 inhibitor + LPS group, RAW264.7 cells were simultaneously incubated with LPS and 100 nm miR155 inhibitor; RAW264.7 cells in the CGS + miR155 inhibitor + LPS group were co‐incubated with CGS21680, miR155 inhibitor, and LPS. Following a 24 h incubation period, both the cells and the culture supernatant were collected for subsequent assays.

Experiment for TGF‐β1 intervention: Primary mHSCs were isolated according to a previously established protocol [60]. In brief, in situ liver perfusion was conducted sequentially using EGTA, 0.4 mg mL^−1^ pronase E, and 0.5 mg mL^−1^ collagenase D, followed by treatment with pronase E and collagenase IV solution. Subsequently, parenchymal cells were removed through nylon net filtration and centrifugation. Density gradient centrifugation was then performed on the nonparenchymal supernatant to isolate mHSCs. The purity of mHSCs was assessed using the autofluorescence of vitamin A droplets. mHSCs were cultured in high‐glucose DMEM medium (containing 10% FBS, 1% penicillin/streptomycin, and 1% HEPES) until confluence reached 80%. To evaluate the synergistic effect of inosine and miR155 inhibitor on anti‐fibrosis, mHSCs were divided into five groups: DMSO, TGF‐β1, Inosine + TGF‐β1, miR155 inhibitor + TGF‐β1, and Inosine + miR155 inhibitor + TGF‐β1 groups. In accordance with the experimental design, mHSCs were subjected to treatment with either DMSO, TGF‐β1 (5 ng mL^−1^) alone, or TGF‐β1 in combination with inosine, miR155 inhibitor, or both inosine and miR155 inhibitor for 24 h. Finally, the mHSCs were collected for further analysis.

### 5.4 *Bifidobacterium pseudolongum* (*B.p*) Culture

*B.p* was acquired from BeNa Culture Collection (Cat#: BNCC135158, Shanghai, China). *B.p* was cultured with BSM broth medium containing 0.5‰ cysteine hydrochloride, 1‰ Tween‐80, 0.3% potassium dihydrogen phosphate, 0.5% yeast extract, 0.5% beef extract, 1% sodium ascorbate, 1% pancreatic digest of casein, and 1% glucose under anaerobic conditions at 37°C for 24 h. The medium was then subjected to centrifugation (3000 rpm for 10 min at 4°C), sterile PBS washing, and resuspension to obtain a 1 × 10^9^ CFU/200 µL *B.p* suspension.

### 5.5 Histological Analysis

As described in our previous studies [61, 62], fresh colon, liver, spleen, kidney, heart, and lung tissues were fixed using 4% paraformaldehyde, dehydrated, paraffin‐embedded, and sectioned into 4 µm slices. According to the manufacturer's instructions, the colon sections were stained with HE using an HE staining kit (Cat#: C0105S, Beyotime, Shanghai, China), alcian blue staining (AB) using an AB staining kit (Cat#: BES‐248SS, BIOESN, Shanghai, China), and periodic acid‐Schiff staining (PAS) using a PAS staining kit (Cat#: C0142M, Beyotime, Shanghai, China), respectively. The liver sections were stained with HE and picrosirius red (PSR). PSR staining was performed using a PSR staining kit (Cat#: AG1470, ACMEC, Shanghai, China). Spleen, kidney, heart, and lung tissues were stained with HE and Masson. Masson staining was performed using a Masson staining kit (Cat#: AG1340, ACMEC, Shanghai, China). Micrographs of the sections were acquired using a Leica M200 Microscope equipped with a Leica DFC320 Digital camera. A double‐blinded method was adopted to count the number of AB^+^ and PAS^+^ goblet cells per crypt, while the relative area of PSR and Masson was determined and calculated using ImageJ software (version 2.0.0).

### 5.6 IHC

IHC staining was performed following the standard procedures. In brief, the embedded colonic and liver tissues were cut into 2‐µm sections and washed using PBS solution with 1% Tween 20 (PBST). Subsequently, the sections were blocked for 1 h using 5% normal donkey serum at room temperature. The colon sections were then incubated with rabbit ZO‐1 primary antibody (Cat#: AF5145, Affinity) at dilution ratio of 1:200 at 4°C overnight, while liver sections were incubated with the following primary antibodies: Anti‐α‐SMA antibody (1:500 dilution, Cat#: A1011, Abclonal), anti‐F4/80 antibody (1:500 dilution, Cat#: 70076, CST), and anti‐CD86 antibody (1:100 dilution, Cat#: 19589S, CST). After PBST washing, the sections were incubated with goat anti‐rabbit secondary antibody (1:400 dilution, Cat#: 205718, Abcam). DAB‐Chromogen was used for the visualization of antibody binding. A Leica M200 Microscope equipped with a Leica DFC320 Digital camera was used for image acquisition, while the ratio of positive area was calculated using ImageJ software.

### 5.7 TUNEL Assay

The apoptosis of colonic cells was detected using an In Situ Cell Apoptosis Detection Kit according to the manufacturer's Instructions (Cat#: BC2100, BASMEDTSCI, Wuhan, China). Colonic tissues were fixed, embedded, and sectioned as described in the Histological analysis. Subsequently, the deparaffinized sections were incubated with 0.1% Triton X‐100 containing 0.01% sodium citrate for 10 min at room temperature. After washing thrice with PBS, sections were incubated in TUNEL reaction mixture at 37°C for 1 h, followed by incubation with DAPI solution (0.05 µg µL^−1^) for 15 min at room temperature in the dark. Fluorescence images were observed and captured using a fluorescence microscope (Leica Microsystems, Nussloch, Germany). The number of TUNEL‐positive cells per field was calculated and analyzed using a double‐blinded method.

### 5.8 TEM

For TEM, small slices of fresh colonic tissues were washed using saline and rapidly fixed with 2.5% glutaraldehyde at room temperature for 1 h. Subsequently, the slices were postfixed with 1% osmium tetroxide at 4°C for 1 h and then underwent a graded series of ethanol dehydration (50%, 70%, 90%, and 100%) followed by Epon resin embedding. Ultrathin sections were prepared and analyzed using TEM Zeiss EM 900 (Milan, Italy).

### 5.9 Flow Cytometry

Detection for M1 polarization in macrophages: After washing thrice with pre‐chilled PBS, RAW264.7 and THP‐1 cells were gently scraped and collected by centrifugation (800 rpm for 5 min) and fixed with pre‐chilled methanol. The cells were then incubated with FITC‐labeled F4/80 (Cat#: 16911, Species Reactivity: Human/Mouse, Abcam) and PE‐labeled CD86 (Cat#: MHCD8604, Species Reactivity: Human/Mouse, Thermo Fisher) for 20 min in the dark. Following incubation, the cells were washed with PBS, resuspended, and subsequently labeled for detection using a BD FACSAria III cell sorter system (Becton–Dickinson, Tokyo, Japan).

### 5.10 Detection for Apoptosis of Hepatocytes

The apoptosis ratio of AML12 cells was determined using an Annexin V‐FITC/PI Apoptosis Detection Kit according to the manufacturer's Instructions (Cat#: 40302ES08, Yeasen, Shanghai, China). In brief, AML12 cells were collected by centrifugation after being washed twice with pre‐chilled PBS. Then, cells were resuspended in 100 µL of 1 × binding buffer. Subsequently, 5 µL of Annexin V‐FITC and 10 µL of PI staining solution were supplemented to suspension. After 15 min of incubation in the dark at room temperature, 400 µL of 1 × binding buffer was added to the cells, and apoptosis was detected using a BD FACSAria III cell sorter system. Cells positive for both Annexin V‐ FITC and PI (FITC^+^ PI^+^) were indicative of being dead or in late apoptosis.

### 5.11 Enzyme‐Linked Immunosorbent Assay (ELISA)

To evaluate systemic inflammation, cardiac blood samples were collected from the mice and allowed to stand for 1 h at room temperature to separate the serum by centrifugation (3000 rpm for 10 min at 4°C). The serum levels of inosine, LPS, TNF‐α, IL‐6, and IL‐1β were determined using ELISA kits according to the manufacturer's Instructions. To determine the inosine level in the liver, the extracts of liver tissue were obtained by grinding, PBS blending, and centrifugation. The inosine level in supernatant was then determined using an ELISA kit. The inflammatory cytokines in the culture supernatant of RAW264.7 cells, as well as liver function indexes ALT and AST in the culture supernatant of AML12 cell, were also measured using ELISA kits. The ELISA kits for inosine (Cat#: MM‐45192M1), LPS (Cat#: MM‐0634M1), TNF‐α (Cat#: MM‐0132M1), IL‐6 (Cat#: MM‐0163M1), ALT (Cat#: MM‐44115M1), and AST (Cat#: MM‐44625M1) were purchased from Jiangsu Meimian Industrial Co., Ltd., while the ELISA kit for IL‐1β (Cat#: MB‐2776A) was purchased from Jiangsu Meibiao Biotechnology Co., Ltd. In addition, the levels of inflammatory cytokines in the culture supernatant of THP‐1 cells were determined using the corresponding ELISA kits: TNF‐α (Cat#: MM‐0122H2), IL‐6 (Cat#: MM‐0049H2), and IL‐1β (Cat#: MM‐0181H2). The optical density (OD) values were measured using an iMark microplate reader at 450 nm wavelength (Bio‐Rad, CA, USA). Finally, the concentration of these cytokines was calculated based on the standard curves.

Moreover, serum biochemical indexes, including ALT, AST, UREA, CREA, LDH, and CK, were measured using an automatic blood biochemical analyzer (Hitachi, Tokyo, Japan).

### 5.12 Real‐Time Quantitative PCR (RT‐qPCR)

Total RNA of colonic and liver tissues, RAW264.7 cells, and THP‐1 cells was isolated using the RNAiso Plus reagent according to the manufacturer's Instructions (Cat#: T9108, TaKaRa, Dalian, China). The quality and quantity of total RNA were measured using a NanoDrop 2000 spectrophotometer (Thermo Scientific, MA, USA). For mRNA, 1 µg of total RNA per sample was reverse transcribed to complementary DNA (cDNA) using the Hifair IIIfirst Strand cDNA Synthesis SuperMix (gDNA digester plus) (Cat#: 11141ES60, Yeasen, Shanghai, China) and subsequently amplified using the Hieff UNICON Universal Blue qPCR SYBR Green Master Mix (Cat#: 11184ES08, Yeasen). For miRNA, the Hifair III first Strand cDNA Synthesis Kit (gDNA digester plus) (Cat#: 11139ES60, Yeasen) was used for reverse transcription, and the resulting cDNA was amplified using the Hieff miRNA Universal qPCR SYBR Master Mix (Cat#: 11170ES08, Yeasen). The relative expression of target genes was calculated using the 2^−ΔΔCt^ method. β‐actin served as the internal reference gene for mRNA, while U6 was used as the reference gene for miR155. The corresponding primers of the target genes are listed in Table S1.

### 5.13 Detection of Fecal *B.p*. Abundance

Total DNA was isolated from fecal samples using a MolPure Stool DNA Kit according to the manufacturer's Instructions (Cat#: 18820ES08, Yeasen). The quality and quantity of the extracted fecal DNA were assessed using a NanoDrop 2000 spectrophotometer. DNA samples were amplified using the Hieff UNICON Universal Blue qPCR SYBR Green Master Mix (Cat#: 11184ES08, Yeasen) on a LightCycler 480 instrument (Roche, Switzerland). The relative abundance of *B.p* was calculated by normalizing to Bacterial 16S rDNA using the 2^−ΔΔCt^ method. The corresponding primers of the target genes are listed in Table S1.

### 5.14 WB

As described in our previous studies [63, 64, 65], total protein of colonic and liver tissues, as well as RAW264.7, THP‐1, and AML12 cells, was extracted with RIPA lysis solution containing 1% phosphatase inhibitor cocktail (Cat#: B15001, Selleck) and 1% protease inhibitor cocktail (Cat#: GK10010, GLPBIO). The concentration of total protein was determined using a BCA Protein Assay Kit (Cat#: BCA, Thermo Scientific). The protein samples were then separated using 12% SDS‐PAGE gels and electrophoretically transferred to 0.22 µm PVDF membranes. After blocking with 5% skim milk for 2 h, the membranes were incubated with specific primary antibodies at 4°C overnight. The membranes were then incubated with the corresponding HRP‐conjugated secondary antibodies. Lastly, protein blots were visualized using a Tanon‐4200 imaging system (Tiangen Biotech, Beijing, China). The primary and secondary antibodies were listed as follows: Anti‐F4/80 antibody (Cat#: 29414‐1‐AP, 1:1000 dilution, Proteintech), anti‐CD86 antibody (Cat#: 19589, 1:1000 dilution, CST), anti‐SOCS1 antibody (Cat#: BD‐PT4362, 1:1000 dilution, Biodragon), anti‐phospho NF‐κB antibody (Cat#: 3033, 1:1000 dilution, CST), anti‐NF‐κB antibody (Cat#: 8242, 1:1000 dilution, CST), anti‐α‐SMA antibody (Cat#: A1011, 1:1000 dilution, Abclonal), anti‐Col1a1 antibody (Cat#: A1352, 1:1000 dilution, Abclonal), anti‐ZO‐1 antibody (Cat#: AF5145, 1:1000 dilution, Affinity), anti‐Occludin antibody (Cat#: 91131, 1:1000 dilution, CST), anti‐Claudin‐5 antibody (Cat#: 29767‐1‐AP, 1:1000 dilution, Proteintech), anti‐A2AR antibody (Cat#: bs‐1456R, 1:1000 dilution, Bioss), anti‐β‐actin antibody (Cat#: 66009‐1‐Ig, 1:2000 dilution, Proteintech), HRP‐conjugated Goat anti‐Rabbit IgG (Cat#: 31460, 1:5000 dilution, Invitrogen), and HRP‐conjugated Goat anti‐Mouse IgG (Cat#: C31430100, 1:5000 dilution, Invitrogen).

### 5.15 16S rRNA Sequencing

On day 29, fecal samples were collected with sterile metabolic cages, and the fecal microbiota DNA was isolated using a MolPure Stool DNA Isolation Kit (Cat#: 18820ES70, Yeasen). The integrity of bacterial DNA was determined using agarose gel electrophoresis, while the quality and concentration of DNA samples were measured with a NanoDrop 2000 spectrophotometer (Thermo Scientific, MA, USA). The V3‐V4 hypervariable region of bacterial 16S rRNA gene was amplified with primer pairs 338F (5’‐ACTCCTACGGGAGGCAGCAG‐3’) and 806R (5’‐GGACTACHVGGGTWTCTAAT‐3’). The purified amplicons were used to construct cDNA library with a NEXTFLEX Rapid DNA‐Seq kit (Bioo Scientific, TX, USA), and the subsequent sequencing was performed using an Illumina NextSeq 2000 PE300 Platform. The raw data were then filtered and merged using the FLASH software to obtain high‐quality clean tags. OTUs with a 97% similarity cutoff were clustered by USEARCH software (version 11). Lastly, the taxonomic annotation of each OTU representative sequence was performed using the RDP classifier (version 11.5) by mapping to the Silva 16S rRNA Gene Database with a confidence coefficient of 0.7.

### 5.16 Untargeted Metabolomics

To extract fecal metabolites, fecal samples (100 mg per sample) were thoroughly ground and mixed with 800 µL of 80% methanol solution. The supernatant was separated using high‐speed centrifugation. Next, 100 µL of the supernatant was mixed with 400 µL of methanol‐acetone solution (1:1 v/v). The mixture was then subjected to vortexing, low‐temperature ultrasound, quiescence, and centrifugation to extract supernatant. Subsequently, the supernatant was blow‐dried with nitrogen and redissolved in a 50% acetonitrile solution. After quiescence and centrifugation, the obtained pure supernatant was analyzed using liquid chromatography‐mass spectrometry (LC/MS) to detect metabolites using a UHPLC‐Q Exactive HF‐X system equipped with an ACQUITY UPLC HSS T3 column (100 × 2.1 mm inner diameter, 1.8 µm; Waters, Milford, USA). The raw data were imported and analyzed using Progenesis QI (Waters, Milford, USA). Metabolites were identified by mapping to the HMDB (http://www.hmdb.ca/), Metlin (https://metlin.scripps.edu), and the self‐compiled Majorbio database (MJDB) of Majorbio Biotechnology Co., Ltd. (Shanghai, China).

### 5.17 RNA Sequencing

Total RNA was isolated from colonic tissues with a TRIzol RNA Extraction Kit (Invitrogen, USA) according to the manufacturer's Instructions. The quantity and purity of RNA samples were determined using a NanoDrop 2000 spectrophotometer (Thermo Scientific, MA, USA), while the RNA integrity was assessed using agarose gel electrophoresis. RNA samples meeting the following requirements were adopted for further RNA sequencing: Total RNA amount ≥ 1 µg, concentration ≥ 30 ng µL^−1^, RQN > 6.5, OD260/280 = 1.8–2.2. The RNA samples were sent to Shanghai Majorbio Bio‐pharm Biotechnology Co., Ltd. (Shanghai, China) for RNA purification, reverse transcription, library construction, and sequencing. The obtained raw reads underwent trimming and quality control to filter high‐quality clean data using the Fastp software (https://github.com/OpenGene/fastp). The clean data were then aligned to the reference genome to identify corresponding genes using the HiSat2 software (http://ccb.jhu.edu/software/hisat2/index.shtml). Subsequently, the expression level of each transcript was quantified using RSEM software (http://deweylab.github.io/RSEM/) and the DEGs were screened using DEGseq2 software (http://bioconductor.org/packages/stats/bioc/DESeq2/) according to the following parameters: Log2FC ≥ 1 or ≤ ‐1, *P*‐value ≤ 0.05. Lastly, GO enrichment and GO annotation analyses were performed on DEGs using the Majorbio Cloud Platform (https://majorbio.com).

### 5.18 Kinetics of Inosine Adsorption by NPs and Characterization

To evaluate the adsorption of inosine to NPs, 5 mg of inosine and 500 mg of NPs were dispersed in 100 mL of PBS to form suspensions. These suspensions were then vortexed for 120 h at 200 rpm at temperatures of 4, 25, and 37°C, respectively. Suspension samples were respectively collected at the following time points: 0, 6, 12, 24, 48, 72, 96, and 120 h. The absorption spectra of the samples were measured using the GENESYS 10S UV–Vis Spectrophotometer (Thermo Scientific, MA, USA), and the concentration of inosine was calculated based on the standard curve. After 120 h of incubation with inosine, the particle size distribution and zeta potential of NPs were assessed using a Malvern zetasizer (Malvern, UK). The surface morphological character of NPs was examined using a scanning electron microscope (Hitachi, Japan), and the surface functional groups were determined using a Fourier Transform Infrared Spectrometer (FTIR, TENSOR 27 Bruker plc, USA).

To further investigate the interactions between *B.p* and NPs, 500 mg of NPs were incubated in 100 mL of either control supernatant or *B.p* supernatant. After vortexing for 120 h at 37°C, the particle size distribution, zeta potential, surface morphological character, and surface functional groups of NPs were determined as described above.

### 5.19 Statistical Analysis

Data were analyzed using GraphPad Prism software (version 9.0.0) and presented as mean ± SEM. Student's t‐test or Wilcoxon rank‐sum test was used for comparison between the two groups, while one‐way ANOVA with post hoc Tukey test was employed for comparisons among multiple groups. All experiments were repeated at least three times. *p‐*value ˂ 0.05 was considered statistically significant (*n* ≥ 3, *p‐value ˂ 0.05, **p‐value ≤ 0.01).

## Author Contributions

J.S. and Q.W.: conceptualization, resources, funding acquisition and project administration; K.Z.: investigation, methodology and writing – original draft; Y.C. and J.W.: data curation, formal analysis and software; J.Y. and L.C.: supervision and validation; A.Z. and Q.L.: visualization; J.D. and N.Z.: writing – review & editing. All authors contributed to manuscript revision, read, and approved the submitted version.

## Conflicts of Interest

The authors declare no conflicts of interest.

## Supporting information




**Supporting File**: advs76423‐sup‐0001‐SuppMat.docx.

## Data Availability

The data used to support the findings of this study are available from the corresponding authors upon reasonable request.
