# Supplementary material for: Bifidobacterium Pseudolongum‐Derived Inosine Mitigates Polystyrene Nanoplastics‐Induced Hepatic Injury by Inhibiting the Polarization of M1 Macrophages
Source: Adv Sci (Weinh). 2026 Jul 6;13(54):e22510. doi: 10.1002/advs.202522510 (PMC13335968; doi:10.1002/advs.202522510)
Supplement: Supplementary file 1 [file ADVS-13-e22510-s001.docx]

Supporting Information

***Bifidobacterium pseudolongum-*Derived Inosine Mitigates Polystyrene Nanoplastics-Induced Hepatic Injury by Inhibiting the Polarization of M1 Macrophages**

*Kaikai Zhang^1, 2^, Yuchuan Chen^1^, Jiayuan Wan^1^, Jianzheng Yang^3^, Lijian Chen^3^, Qingyuan Li^3^, Anding Zhou^3^, Nian Zhou^3^, Jiuyang Ding^3^, Qi Wang^3*^, Jian Sun^1*^*

K. Zhang, Y. Chen, J. Wan, J. Sun

^1^Department of Infectious Diseases, Nanfang Hospital, Southern Medical University; State Key Laboratory of Multi-organ Injury Prevention and Treatment; Key Laboratory of Infectious Diseases Research in South China, Ministry of Education; Guangdong Provincial Key Laboratory for Prevention and Control of Major Liver Diseases; Guangdong Provincial Clinical Research Center for Viral Hepatitis; Guangdong Institute of Hepatology; Guangdong Provincial Research Center for Liver Fibrosis Engineering and Technology

Southern Medical University

Guangzhou 510515, China.

E-mail address: sunjian@smu.edu.cn

K. Zhang

^2^Department of Gastroenterology, Shunde Hospital of Southern Medical University,

Southern Medical University

Foshan 528399, China.

J. Yang, L. Chen, Q. Li, A. Zhou, N. Zhou, J. Ding, Q. Wang

^3^Guangzhou Key Laboratory of Forensic Multi-Omics for Precision Identification, School of Forensic Medicine

Southern Medical University

Guangzhou 510515, China.

E-mail address: wangqi1980@smu.edu.cn

**K. Zhang, Y. Chen, J. Wan and J. Yang** contributed equally to this work and share first authorship.

**Supplementary Tables**

**Supplementary Table 1**

**The sequence of primers for RT-qPCR**

| **Gene** | **Forward** | **Reverse** |
| --- | --- | --- |
| **TNF-α** | 5'-TAGCCAGGAGGGAGAACAGA-3' | 5'-TTTTCTGGAGGGAGATGTGG-3' |
| **IL-6** | 5'-ATTTCCTCTGGTCTTCTGG-3' | 5'-CTGGCTTTGTCTTTCTTGT-3' |
| **IL-1β** | 5'-GAAATGCCACCTTTTGACAGTG-3' | 5'-TGGATGCTCTCATCAGGACAG-3' |
| **β-actin** | 5'-CATTGCTGACAGGATGCAGAAGG-3' | 5'-TGCTGGAAGGTGGACAGTGAGG-3' |
| **Mmu-**  **miR155** | 5'-CGCGGCCTTAATGCTAATTGTGA-3' | 5′-ATCCAGTGCAGGGTCCGAGG-3′ |
| **Mmu-U6** | 5'-CTCGCTTCGGCAGCACATATACT-3' | 5′-ACGCTTCACGAATTTGCGTGTC-3′ |
| **Hsa-**  **miR155** | 5'-TTAATGCTAATCGTGATAGGGGTT-3' | 5'-CAGTGCAGGGTCCGAGGTA-3' |
| **Hsa-U6** | 5'-CTCGCTTCGGCAGCACA-3' | 5'-AACGCTTCACGAATTTGCGT-3' |
| ***B.p*** | 5'-ACAGGTCGTCAGCGAAGATT-3' | 5'-GTGGAACCCTGCGGATACTC-3' |
| **Bacterial 16S rDNA** | 5'-CCTACGGGAGGCAGCAG-3' | 5'-ATTACCGCGGCTGCTGG-3' |

**Supplementary Figures**

**Supplementary Figure 1**

The dose- and size-dependent influences of plastic particle on liver weight, *B.p* abundance and inosine concentration. (A) Alterations in liver weight across the PBS, L-NPs, H-NPs, and H-MPs groups. (B) Alterations in proportions of probiotics and pathogens between the PBS and NPs groups at the species level. (C) Alterations in fecal *B.p* abundance across the groups. (D) Alterations in inosine levels in the serum and liver across the groups. All data were analyzed using one-way ANOVA with post hoc Tukey and expressed as mean ± SEM, **P-value* ˂ 0.05, ***P-value* ≤ 0.01, N.S: *P-value* ≥ 0.05, *n* ≥ 3.

**Supplementary Figure 2**

H-NPs exposure decreased serum and liver concentrations of inosine, and expression level of protein A2AR in liver. (A) Alterations in inosine levels in the serum and liver between the PBS and H-NPs groups. (B) WB analysis of A2AR expression in the liver, normalized to β-actin. All data were analyzed using unpaired Student’s t-test and expressed as mean ± SEM, **P-value* ˂ 0.05, ***P-value* ≤ 0.01, *n* ≥ 3.

**Supplementary Figure 3**

Evaluation of the toxic effects of H-NPs on heart, kidney, spleen, and lung. (A) Alterations in serum levels of LDH and CK between the PBS and H-NPs groups. (B) Alterations in serum levels of UREA and CREA between the two groups. (C) Representative images of HE and Masson staining in the heart, kidney, spleen, and lung, and the corresponding quantification of Masson staining. Scale bar = 200 μm. All data were analyzed using unpaired Student’s t-test and expressed as mean ± SEM, **P-value* ˂ 0.05, ***P-value* ≤ 0.01, *n* ≥ 3.

**Supplementary Figure 4**

(A) Alterations in serum and liver levels of inosine across the PBS, H-NPs, *B.p*, *B.p* + H-NPs, Inosine, and Inosine + H-NPs groups. All data were analyzed using one-way ANOVA with post hoc Tukey and expressed as mean ± SEM, **P-value* ˂ 0.05, ***P-value* ≤ 0.01, N.S: *P-value* ≥ 0.05, *n* ≥ 3.

**Supplementary Figure 5**

Equivalence assessment of protective effects between *B.p* colonization and inosine supplementation. (A) Alterations in inflammatory cytokines: LPS, TNF-α, IL-6, and IL-1β levels in the serum across the PBS, H-NPs, *B.p* + H-NPs, and Inosine + H-NPs groups. (B) Alterations in liver function indexes: ALT and AST across the groups. (C, D) Alterations in relative positive area of PSR, α-SMA, F4/80, and CD86 staining. All data were analyzed using one-way ANOVA with post hoc Tukey and expressed as mean ± SEM, **P-value* ˂ 0.05, ***P-value* ≤ 0.01, N.S: *P-value* ≥ 0.05, *n* ≥ 3.

**Supplementary Figure 6**

(A) Alterations in serum and liver levels of inosine across the WT-PBS, WT-H-NPs, miR155^-/-^-PBS, and miR155^-/-^-H-NPs groups. All data were analyzed using one-way ANOVA with post hoc Tukey and expressed as mean ± SEM, **P-value* ˂ 0.05, ***P-value* ≤ 0.01, N.S: *P-value* ≥ 0.05, *n* ≥ 3.

**Supplementary Figure 7**

The miR155 inhibitor ameliorated LPS-induced polarization of M1 macrophages *in vitro*. (A) Representative analysis of the percentage of F4/80^+^ CD86^+^ RAW264.7 cells detected using flow cytometry in the LPS, CGS21680 + LPS, miR155 inhibitor + LPS, and CGS21680 + miR155 inhibitor + LPS groups. (B) RT-qPCR analysis for the expression of miR155 in RAW264.7 cells; U6 was used as an internal control. (C) WB analysis of A2AR, SOCS1, p-NF-κB, and NF-κB expression in RAW264.7 cells, normalized to β-actin. (D) Concentrations of the inflammatory cytokines TNF-α, IL-6, and IL-1β in culture supernatants across the groups. (E) WB analysis of Col1a1 and α-SMA expression in mHSCs cells across the DMSO, TGF-β1, Inosine + TGF-β1, miR155 Inhibitor + TGF-β1, and Inosine + miR155 Inhibitor + TGF-β1 groups. β-actin was served as a loading control. All data were analyzed using one-way ANOVA with post hoc Tukey and expressed as mean ± SEM, **P-value* ˂ 0.05, ***P-value* ≤ 0.01, N.S: *P-value* ≥ 0.05, *n* ≥ 3.
